# Prediction of bacterial protein–compound interactions with only positive samples

**DOI:** 10.1093/bioinformatics/btag067

**Published:** 2026-02-18

**Authors:** Ki-Hwa Kim, Avinash Yaganapu, Sai Kosaraju, Aashish Bhatt, Yun Lyna Luo, Sai Phani Parsa, Juyeon Park, Hyun Lee, Jun Hyuck Lee, Tae-Jin Oh, Mingon Kang

**Affiliations:** Genome-Based BioIT Convergence Institute, Asan, 31460, Republic of Korea; Department of Computer Science, University of Nevada, Las Vegas, Las Vegas, NV, 89154, United States; Department of Computer Science, California State Polytechnic University, Pomona, CA, 91768, United States; Department of Biotechnology and Pharmaceutical Sciences, Western University of Health Sciences, Pomona, CA, 91766, United States; Department of Biotechnology and Pharmaceutical Sciences, Western University of Health Sciences, Pomona, CA, 91766, United States; Department of Computer Science, University of Nevada, Las Vegas, Las Vegas, NV, 89154, United States; Division of Computer Science and Engineering, Sun Moon University, Asan, 31460, Republic of Korea; Division of Computer Science and Engineering, Sun Moon University, Asan, 31460, Republic of Korea; Division of Life Sciences, Korea Polar Research Institute, Incheon, 21990, Republic of Korea; Genome-Based BioIT Convergence Institute, Asan, 31460, Republic of Korea; Department of AI Biomedical Engineering, Graduate School, Sun Moon University, Asan, 31460, Republic of Korea; Department of Pharmaceutical Engineering and Biotechnology, Sun Moon University, Asan, 31460, Republic of Korea; Department of Computer Science, University of Nevada, Las Vegas, Las Vegas, NV, 89154, United States

## Abstract

**Motivation:**

Prediction of Compound–Protein Interactions (CPI) in bacteria is crucial to advance various pharmaceutical and chemical engineering fields, including biocatalysis, drug discovery, and industrial processing. However, current CPI models cannot be applied for bacterial CPI prediction due to the lack of curated negative interaction samples.

**Results:**

We propose a novel Positive-Unlabeled (PU) learning framework, named BIN-PU, to address this limitation. BIN-PU generates pseudo positive and negative labels from known positive interaction data, enabling effective training of deep learning models for CPI prediction. We also propose a weighted positive loss function that weights to truly positive samples. We have validated BIN-PU coupled with multiple CPI backbone models, comparing the performance with the existing PU models using bacterial cytochrome P450 (CYP) data. Extensive experiments demonstrate the superiority of BIN-PU over the benchmark models in predicting CPIs with only truly positive samples. Furthermore, we have validated BIN-PU on additional bacterial proteins obtained from literature review, human CYP datasets, and uncurated data for its reproducibility. We have also validated the CPI prediction for the uncurated CYP data with biological and biophysical experiments. BIN-PU represents a significant advancement in CPI prediction for bacterial proteins, opening new possibilities for improving predictive models in related biological interaction tasks.

**Availability and implementation:**

The source code and data are available at https://github.com/datax-lab/CYP.

## 1 Introduction

Accurate prediction of Compound–Protein Interactions (CPI) in bacteria is essential in discovering diverse chemical properties and understanding intermediate mechanisms of biosynthesis and biodegradation (Singh Nigam 2013, Darbandi *et al.* 2024). Bacterial enzymes act as biocatalysts that react with compounds and are widely utilized in sectors including agriculture, chemical industry, food, textiles, pharmaceuticals, cosmetics, and bioremediation (Leibbrandt 1996, Darbandi *et al.* 2024). Bacterial proteins metabolize a wide spectrum of endogenous and exogenous compounds, enhancing the efficiency of biocatalyst processes that selectively interact with bioactive compounds (Ben Akacha and Gargouri 2015, Liu and Kokare 2023). However, characterizing biological interactions between proteins and compounds remains extremely challenging due to the complexity of protein networks, heterogeneity in protein behavior across environments, and high substrate selectivity (Silver 2011, Typas and Sourjik 2015, Hu *et al.* 2023).

Deep learning-based CPI models have been explored using rich reference databases, particularly focusing on human proteins and compounds. These models learn relevant features directly from protein and compound data, enhancing their capability to discover complex interactions—unlike traditional machine learning approaches that rely on predefined features, such as kernel-based SVMs (Bleakley and Yamanishi 2009, Cheng *et al.* 2012, Tabei and Yamanishi 2013, Lapins *et al.* 2018), graph data mining (Yamanishi *et al.* 2008), and tree-based classifiers (Shi *et al.* 2019). Moreover, non-linear relationships and hierarchical representations are effectively captured by deep learning models, which are crucial for understanding CPIs. Current state-of-the-art deep learning CPI models utilize Graph Neural Networks (GNNs), Convolutional Neural Networks (CNNs), and Transformers. For instance, CNN-GNN encodes protein sequences using CNN and compounds using GNN, followed by feature integration to predict their interactions (Tsubaki *et al.* 2019). TransformerCPI uses a transformer-based framework with an encoder-decoder structure to extract low-level representations of proteins and compounds (Chen *et al.* 2020). SSNet incorporates protein secondary structure databases along with CNN and GNN-based representations for both proteins and compounds (Verma *et al.* 2021). MulinforCPI integrates protein-language-model embeddings with graph-based compound representations to improve CPI prediction performance (Nguyen *et al.* 2023). However, all these CPI models require both positive (i.e. known interactions) and negative (i.e. known non-interactions) samples for training, which limits their applicability to bacterial CPI prediction.

Deep learning models for bacterial CPI prediction have seldom been developed, primarily due to the lack of databases containing known non-interactions. In microorganisms, the extremely limited availability of negative samples presents a significant barrier to utilizing current state-of-the-art models that depend on both positive and negative interactions. Several factors contribute to this challenge. First, the complexity and diversity of bacterial species hinder the identification of true negative samples, as interaction likelihood varies across species and environmental contexts. Moreover, experimental designs involving heterologous expression of bacterial proteins may not accurately represent their native environments (Rong *et al.* 2023). Consequently, the absence of inactive samples creates a substantial gap, limiting the development of effective CPI prediction models for bacterial systems.

Positive-Unlabeled (PU) learning presents a promising solution by enabling model training using only positive samples. PU learning typically extends the training set by inferring pseudo labels for likely positive and negative examples derived solely from truly positive data (Bekker and Davis 2020). Generative Adversarial Networks (GANs) have been leveraged in PU settings, where a generator produces synthetic negatives and a discriminator distinguishes between generated and real positives (Hou *et al.* 2018, Hu *et al.* 2021). Alternatively, sample-wise weighting schemes, in which positive samples are assigned greater weights than unlabeled data, to improve classification performance by penalizing misclassification and ensuring consistency through resampling under PU settings (Claesen *et al.* 2015, Cheng *et al.* 2018). Recently, representation-based PU learning approaches have gained attention. For instance, PU-Contrastive introduces a positive-only contrastive objective that learns discriminative representations. PU-Contrastive maximizes the similarity between augmented views of positive samples while separating them from unlabeled samples in the latent space, which improves the separability of likely positive interactions under PU settings (Acharya *et al.* 2024). In parallel, graph-based PU learning methods, such as PU-GNN, extend to graph-structured data by modeling sample dependencies through graph connectivity (Keshavarz and Lakizadeh 2024).

**Figure 1:**
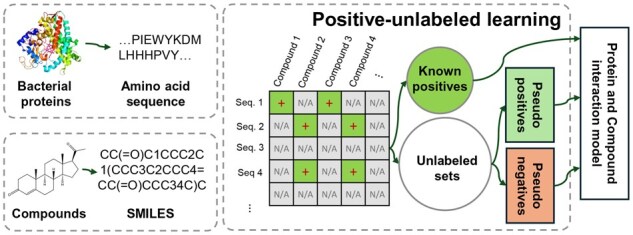
Overview of the study. The proposed PU learning strategy trains deep learning models to predict interactions between bacterial proteins and compounds using only known interaction databases by generating pseudo positives and negatives from unlabeled datasets.

In this study, we propose an effective PU learning framework, BIN-PU (BINning strategy for PU), to predict interactions between bacterial proteins and compounds. BIN-PU generates reliable pseudo positive and negative labels from truly positive data and builds a classifier with a weighted positive loss function to predict CPIs (Fig. 1). We evaluated BIN-PU’s robust predictive performance in multiple settings. First, we assessed BIN-PU coupled with CPI backbone models on the cross-validation dataset, compared to the existing PU strategies. Second, we validated BIN-PU using independent bacterial CYP from the literature and curated human enzymes. Finally, we applied BIN-PU to uncurated bacterial CYPs to explore novel enzyme–substrate interactions. The predicted interactions were experimentally verified through biological assays (HPLC) and biophysical analysis. The details of the BIN-PU framework and its implementation are elucidated in Methods. In summary, our main contributions are: (i) BIN-PU predicts potential interactions between compounds and bacterial proteins using only truly positive samples; (ii) Extensive experiments coupled with CPI backbone models were conducted to validate the effectiveness and advantages of the proposed PU strategy, compared to the current state-of-the-art methods PUCPI (PU learning for CPI identification) (Cheng *et al.* 2018) and PU-Contrastive (Positive Unlabeled Contrastive Learning) (Acharya *et al.* 2024); (iii) We further validated the model using bacterial CYP and substrate data through laboratory assays, integrating both biological validation and molecular docking studies. To the best of our knowledge, this is the first method to predict CPIs for bacterial enzymes using only truly positive dataset.

## 2 Materials and methods

### 2.1 Current PU learning strategy for CPI

PU learning has been explored for CPI prediction to address computational challenges when only positive interactions are available. The only PU learning strategy in CPI is PUCPI (Cheng *et al.* 2018), which primarily focused on human enzyme data. PUCPI trains a binary classifier by treating unlabeled compound–protein pairs as negatives, based on the assumption that most unlabeled interactions do not bind. The method generates unlabeled samples from combinations of truly positive interactions and determines the decision boundary by assigning higher weights to the positive class using a biased SVM. However, PUCPI has several limitations: (i) it assumes that most unlabeled samples are negative, which can bias the classifier; (ii) manually up-weighting positive samples is difficult to tune in highly imbalanced positive-only settings; (iii) the absence of reliable negatives complicates evaluation; and (iv) PUCPI was validated using a limited number of positive samples and external data, limiting assessment of false-negative predictions.

### 2.2 The proposed PU learning strategy

**Figure 2:**
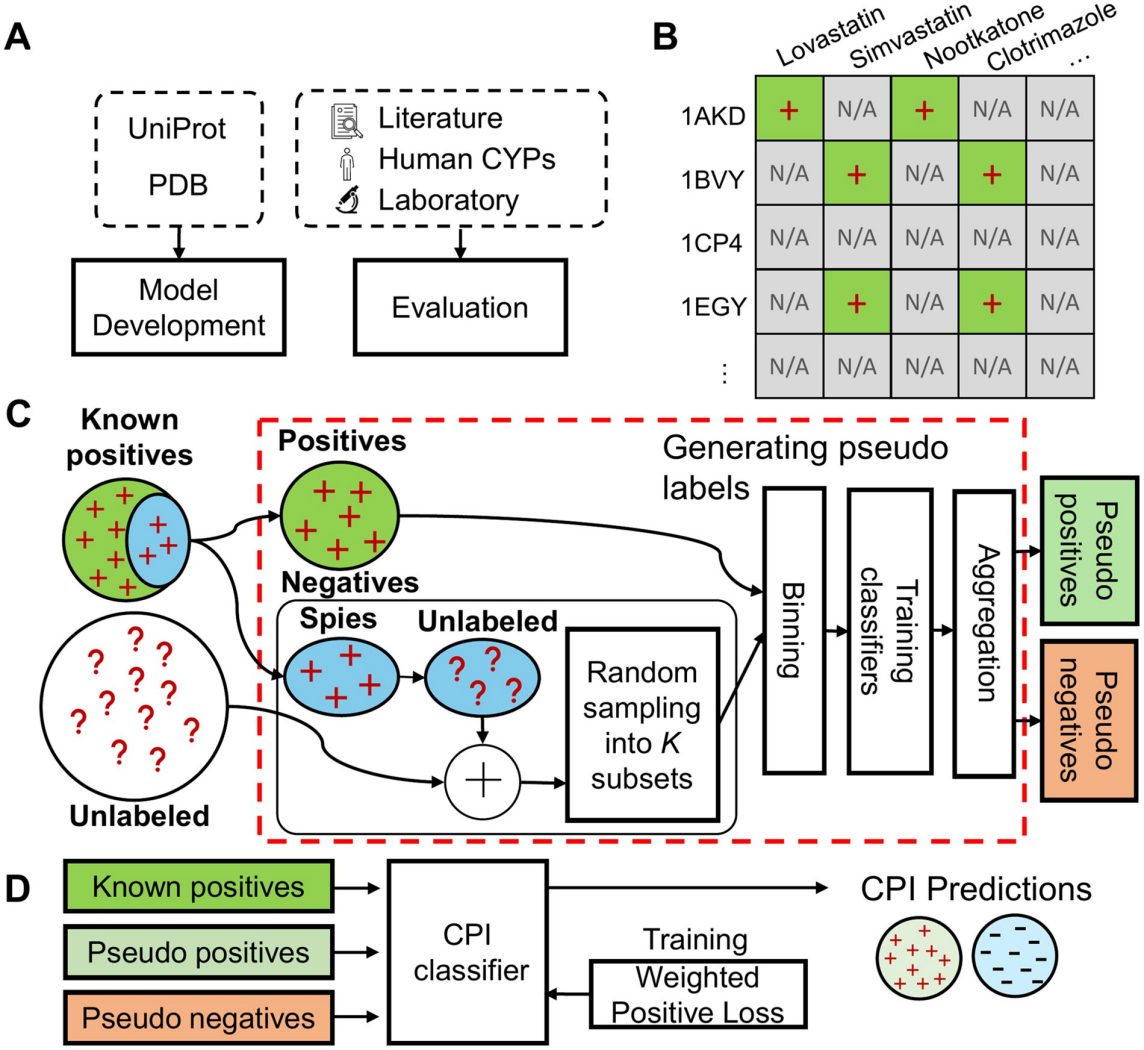
Overview of the proposed framework. (A) We collected datasets from Swiss-Prot and Protein Data Bank (PDB) for model development and assessed the model using literature, human data, and laboratory results for model evaluation. (B) Unlabeled data is generated from known positive samples. (C) A binning-based BIN-PU strategy generates pseudo labels from multiple classifiers trained on truly positive and unlabeled data. (D) Finally, the CPI prediction models are trained with an adaptive loss function using these pseudo labels along with known positive samples.

We introduce a novel PU learning strategy, BIN-PU, that addresses the limitations of existing PU learning approaches. It integrates pseudo labeling into a unified learning framework for CPI prediction under positive-only supervision. The overall pipeline of BIN-PU consists of the steps: (i) generating unlabeled data, (ii) generating pseudo labels of interactions between proteins and compounds only from positive data (the key function of BIN-PU), and (iii) training supervised backbone CPI prediction models using the pseudo positive and negative labels (Fig. 2). Unlabeled data is generated from the pairwise combinations between unique proteins and compounds in truly positive data. Then, the proposed model, BIN-PU, generates pseudo labels from the truly positive samples and unlabeled data. Finally, existing backbone CPI prediction models are trained with the generated pseudo positive/negative labels and truly positive samples, along with a new proposed, weighted positive loss function for the PU learning. The details are as follows.

#### 2.2.1 Generating pseudo labels from positive only data

Pseudo positive and negative labels are computationally annotated by BIN-PU. First, pairwise combinations between unique proteins and compounds in truly positive interactions generate unlabeled samples, where truly positive interactions are excluded. The large volume of the unlabeled samples include both likely positive and negative interactions. Multiple bins are created by sampling from the positive and unlabeled interaction data. Due to the scarcity of positive samples and the abundance of unlabeled data, each bin includes a fixed small number of the truly positive samples and large unlabeled data. Each bin trains a binary CPI classifier, where unlabeled data is considered as negative for the model training. Then, unlabeled samples are categorized into pseudo positives and negatives by computing the average prediction scores of the multiple CPI classifiers.

Specifically, given *N* numbers of known positive training samples, $D^{p}=\{C_{i},P_{i},I_{i}^{p};1<i<N\}$, where $C_{i}$ is a SMILES representation of a compound, $P_{i}$ is a protein sequence, and $I_{i}^{p}\in \{1\}$ indicates the interaction between the proteins and compounds from all positives in the given dataset. Unlabeled samples, denoted as $D^{u}=\{C_{i},P_{i},I_{i}^{u}\in \{0\;\text{or}\;1\};1<i<N\}$, are generated by the pairwise combinations of $C_{i}$ and $P_{i}$, excluding known interactions, $I_{i}$. The positive dataset $D^{p}$ is split into two: positive samples for training ($D_{tr}^{p}$) and spy samples for validation ($D_{\mathrm{spy}}^{p}$). $D_{\mathrm{spy}}^{p}$ are added to an unlabeled set, i.e. $D^{u}=D^{u}\cup D_{\mathrm{spy}}^{p}$, which is used for the validation set to assess the BIN-PU’s performance. For training CPI prediction model, $D_{tr}^{p}$ and $D^{u}$ are utilized as positive and negative samples respectively for training, and $D_{\mathrm{spy}}^{p}$ for validating the model.

The negative set $D^{u}$ is randomly partitioned into *K* subsets, $D_{1}^{u},D_{2}^{u},\ldots ,D_{K}^{u}$, where each $D_{k}^{u}$ represents a subset of $D^{u}$ for the *k*th bin. The training set, $D_{k}$, for the *k*th bin is then created by combining $D_{tr}^{p}$ with the *k*th subset of $D^{u}$, as:


(1) $$D_{k}=D_{tr}^{p}\cup D_{k}^{u}.$$

The average posterior probability across all bins are computed as follows:


(2) $$\bar{Pr}=\frac{1}{K}\sum_{k=1}^{K}f(D_{k}),$$

where f(·) denotes the CPI backbone model (e.g. CNN-GNN, TransformerCPI, SSNet, or MulinforCPI), and $f(D_{k})$ are the prediction scores generated by the CPI backbone model. The averaged prediction score across all *K* bins is considered as a pseudo score, denoted as ($\bar{Pr}$), which is to identify high and low confidence samples. The top-ranked interactions (e.g. $\bar{Pr}>\theta_{u}$) are labeled as pseudo positives ($\Psi_{\mathrm{pos}}$), whereas the bottom-ranked samples (e.g. $\bar{Pr}<\theta_{l}$) are labeled as pseudo negatives ($\Psi_{\mathrm{neg}}$). θ is a hyperparameter that determines reliable pseudo-labels. We set $\theta_{u}$ = 0.8 and $\theta_{l}$ = 0.2 in this study. The remains are unlabeled, which are not used for further training.

#### 2.2.2 Spies capture rate

During the generation of pseudo labels, a Spies Capture Rate (SCR) assesses the model’s performance of each bin in identifying the known positives of “spies” from unlabeled dataset. These spies are initially parts of the known positive samples but are set aside for the dual purpose: to contribute to training the model to distinguish known positives from unlabeled sets and to evaluate the model’s capability to correctly identify them as positives. The spies capture rate can be computed by:


(3) $$\mathrm{SCR}=\frac{N_{TS}}{N_{S}},$$

where $N_{TS}$ represents the number of spies predicted as positives, and $N_{S}$ represents the total number of spies. A higher SCR (e.g. close to one) indicates that the model is effective at identifying positive pseudo labels from negatives. However, a limitation of this metric arises when the model incorrectly predicts a large number of unlabeled samples as positive. In such cases, the SCR may appear artificially high, because the model would correctly identify most spy samples as positives. However, labeling the top $\theta_{u}$ and bottom $\theta_{l}$ as pseudo positives and negatives respectively prevent the limitation in our strategy.

#### 2.2.3 Weighted positive loss function for PU learning

Along with the labeled samples of pseudo samples and known positive interactions, a CPI model is trained with adaptive weights. Traditional loss functions (e.g. binary cross entropy) typically minimize classification errors, considering the same weights of the misclassifications in the truly positive samples and pseudo positive samples. To tackle this limitation, we propose a weighted positive loss term that weighs more to known positives for PU learning, ensuring that the model reduces the misclassification to truly positive samples. The proposed loss function consists of two terms, Binary Cross Entropy (BCE) and the weighted positive loss. The weighted positive loss term penalizes the errors of the predictions in truly positive instances, while the BCE term trains the model using entire samples as a binary classifier. The incorporation of the weighted positive loss term with BCE enhances the calibration of the predicted probabilities in the PU learning setting. The proposed loss function is formulated as:


(4) $$L=L_{\text{BCE}}+\lambda L_{\text{WP}},$$

(5) $$L_{\text{BCE}}=-\frac{1}{N}\sum_{i=1}^{N}\left[y_{i}\cdot \;\text{log}({\hat{y}}_{i})+(1-y_{i})\cdot \;\text{log}(1-{\hat{y}}_{i})\right],$$

(6) $$L_{\text{WP}}=\frac{1}{\left|D^{p}\right|}\sum_{i=1}^{\left|D^{p}\right|}{\left(\text{log}({\hat{y}}_{i}+1)-\text{log}(y_{i}+1)\right)}^{2},$$

where *N* represents the combination of truly positive, pseudo positive and negative samples (i.e. $N=|D^{p}|+|\Psi_{\text{pos}}|+|\Psi_{\text{neg}}|$), $y_{i}$ is the true label for instance *i*, ${\hat{y}}_{i}$ is the predicted label of instance *i*, and λ represents a weight coefficient assigned to the positive class during the model training.

## 3 Results

### 3.1 Dataset for model development

In this study, we focus on bacterial CYP, which is a well-known monooxygenase that catalyzes secondary metabolism, such as biosynthesis of steroid hormones. CYP consists of a unique protein structure that contains heme molecules. Heme molecules induce modification of the backbone or side chain of the compound through redox reactions. CYP is a candidate that may interact with diverse compounds for unknown metabolism, due to its characteristics, such as regioselectivity, stereoselectivity, and a broad substrate spectrum. Furthermore, CYP exists in all living things, as it has been isolated from mammals, bacteria, and even viruses.

We collected labeled bacteria data from the two databases of Protein Data Bank (PDB) and UniProtKB/Swiss-Prot (Swiss-Prot). The two databases include proteins, compounds, and their interactions, where proteins and compounds are provided as sequences of amino acids and SMILES strings converted from InChIKey, respectively. We searched the PDB and Swiss-Prot databases using the keywords “Cytochrome P450” and “bacteria.” To enhance the quality of the datasets, we manually examined the dataset. We removed non-CYP proteins and irrelevant proteins and ligands (Note 1, available as supplementary data at *Bioinformatics* online, Tables 1 and 2, available as supplementary data at *Bioinformatics* online). In PDB and Swiss-Prot, we excluded non-binding ligands (e.g. ionic compounds, small molecules, and linkers other than the substrate) and HEM. To label the interactions, we referred to the column of “unique ligands” for each enzyme entry in PDB and “catalytic activity” in Swiss-Prot as truly positive samples. We finally obtained 500 truly positive entries of unique compounds (N=291) and proteins (N=241). Then, we generated unlabeled data by considering the pairwise combinations. The unlabeled data excluded the known interactions (i.e. positive samples), resulting in 69 631 entries that include both likely-positive and negative samples.

**Table 1:** CPI scores predicted by BIN-PU for the five uncurated CYP and the three steroids.^a^

Experiment no.	CYP	Substrate	Biological/biophysical assessment		BIN-PU			
			HPLC	AutoDock Vina^b^	Trans-formerCPI	Mulin-forCPI	CNN-GNN	SSNet
					(θ = 0.47)^c^	(θ = 0.47)	(θ = 0.48)	(θ = 0.46)
Exp. 1	CYP154C9	4-Androstenedione	Pos	Pos [−6.35, C2 (4.07)]	Pos (0.941)^d^	Pos (0.865)	Pos (0.879)	Pos (0.765)
Exp. 2	CYP154C9	Progesterone	Pos	Pos [−7.19, C2 (4.14)]	Pos (0.839)	Pos (0.762)	Neg (0.289)	Neg (0.303)
Exp. 3	CYP154C9	Nandrolone	Pos	Pos [−8.27, C2 (4.49)]	Pos (0.530)	Pos (0.516)	Pos (0.640)	Neg (0.449)
Exp. 4	CYP1047A9	Nandrolone	Pos	Pos [−8.24, C2 (3.91)]	Pos (0.548)	Pos (0.584)	Neg (0.449)	Pos (0.525)
Exp. 5	CYP107G9	Nandrolone	Pos	Pos [−7.66, C2 (4.57)]	Pos (0.759)	Pos (0.880)	Pos (0.763)	Neg (0.478)
Exp. 6	CYP106A4	Nandrolone	Pos	Pos [−8.48, C15 (4.69)]	Pos (0.490)	Pos (0.512)	Neg (0.390)	Neg (0.09)
Exp. 7	CYP106A5	Nandrolone	Pos	Pos [−6.61, C16 (4.82)]	Pos (0.717)	Pos (0.650)	Neg (0.251)	Pos (0.966)
Exp. 8	CYP106A4	Prednisone	Neg	Neg [−6.31, C12 (5.29)]	Neg (0.117)	Neg (0.255)	Neg (0.178)	Pos (0.536)
Exp. 9	CYP106A5	Prednisone	Neg	Neg [−6.15, C16 (5.29)]	Neg (0.140)	Neg (0.330)	Pos (0.461)	Neg (0.112)

a The reactions were validated through biological (HPLC) and biophysical (AutoDock Vina) experiments.

b Binding chance [Vina Score (kcal/mol), Nearest carbon (Dis.Å)].

c The optimal threshold of the discriminant function: positive if the predictive score is higher than θ.

d Predictive scores of the models are in parentheses.

**Table 2:** Results of *in vitro* reactions involving two enzymes (CYP154C9 and CYP106A4) and three electron transport systems using nandrolone as a substrate.

	Fdx–FdR	Pdx–PdR	Iodo-benzene
CYP154C9	Pos	Neg	Neg^a^
CYP106A4	Neg^a^	Neg	Pos

a First confirmed reaction in this study.

### 3.2 Assessment of the predictive performance with cross validation

We evaluated the performance of our proposed BIN-PU against the PU learning benchmark models of PUCPI and PU-Contrastive. We performed two assessments in this cross-validation experiment: (i) assessment of pseudo labels and (ii) evaluation of the performance in a fully supervised learning manner on the test data. First, we generated the pseudo labels from truly positive and unlabeled data and assessed the quality of the pseudo labels on validation data. Then, we trained BIN-PU using the truly positive and reliable pseudo labeled samples coupled with CPI backbone models in a fully supervised manner. We compared the performance of BIN-PU with PUCPI and PU-Contrastive using test data. We considered SSNet (Verma *et al.* 2021), CNN-GNN (Tsubaki *et al.* 2019), TransformerCPI (Chen *et al.* 2020), and MulinforCPI (Nguyen *et al.* 2023) as CPI backbone models for all PU learning models. Detailed implementation and tuning of these models are provided in the Supplementary Materials (Note 2 and 3, available as supplementary data at *Bioinformatics* online).

For the entire experiment, we randomly split the truly positive samples into 60% for training (N=300), 20% for validation (N=100), and 20% for testing (N=100). Then, BIN-PU generated pseudo labels from the unlabeled data of the training. The generated pseudo labeled samples were again split into 60%, 20%, and 20% to aggregate with the truly positive samples in the training, validation, and test group, respectively. The final sample sizes were (N=20,178) for training, (N=6,726) for validation, and (N=6,726) for test on average. We repeated all of the experiments ten times to ensure reproducibility.

For the assessment of the quality of the pseudo labels, we computed the Spies Capture Rate (SCR). We calculated SCR by counting the number of positive predictions among the spy samples in the multiple bins. The SCR scores for BIN-PU coupled with SSNet, CNN-GNN, TransformerCPI, and MulinforCPI were 0.846±0.006, 0.904±0.001, 0.920±0.001, and 0.914±0.002, respectively (Table 3, available as supplementary data at *Bioinformatics* online). BIN-PU with TransformerCPI achieved the highest SCR, which implies that 92% of the spy samples are correctly identified by BIN-PU as positive in the unlabeled data. Note that PUCPI and PU-Contrastive does not generate pseudo labels, and SCR was not compared.

For the assessment of the predictive performance, we computed F1-scores to compare the performance of BIN-PU with PUCPI and PU-Contrastive. For BIN-PU, we empirically optimized hyperparameters using the validation data. Learning rate (i.e. 1e−4), weight decay (i.e. 1e−5), and bin size (20) were optimized to maximize SCR, whereas the optimal weight parameter (λ) in (4) and the threshold for the final discriminant function were obtained by maximizing F1-scores in the validation data (Fig. 1, available as supplementary data at *Bioinformatics* online and Table 4, available as supplementary data at *Bioinformatics* online). We also examined the sensitivity of the thresholds ($\theta_{u}$ and $\theta_{l}$) that determine pseudo positive and pseudo negative samples (Note 3, available as supplementary data at *Bioinformatics* online). Specifically, we evaluated multiple percentile thresholds (top-10%–top-50%) to identify high- and low-confidence samples across all CPI backbone models. BIN-PU demonstrated stable performance across all threshold settings, and the top-20% threshold consistently yielded the highest predictive power (Table 4, available as supplementary data at *Bioinformatics* online). We set $\theta_{u}=0.8$ and $\theta_{l}=0.2$ for all the experiments in this study. For PUCPI, we considered the same CPI backbone models for fair comparison with BIN-PU along with biased SVM that the original PUCPI study used (Supplementary 2, available as supplementary data at *Bioinformatics* online). For PUCPI, we empirically determined the optimal weight for positive samples across all four CPI backbone models. In PUCPI coupled with a biased-SVM, the threshold for the final discriminant function was optimized to maximize F1-scores with the validation data, using the pseudo labels generated by BIN-PU coupled with TransformerCPI. For PU-Contrastive, we optimized the hyperparameters of the temperature parameter (τ) in the contrastive loss, the embedding dimension, and the batch size to maximize F1-scores on the validation data. The training procedures for all four CPI backbone models were provided in the Supplementary (Notes 2 and 3, available as supplementary data at *Bioinformatics* online).

**Figure 3:**
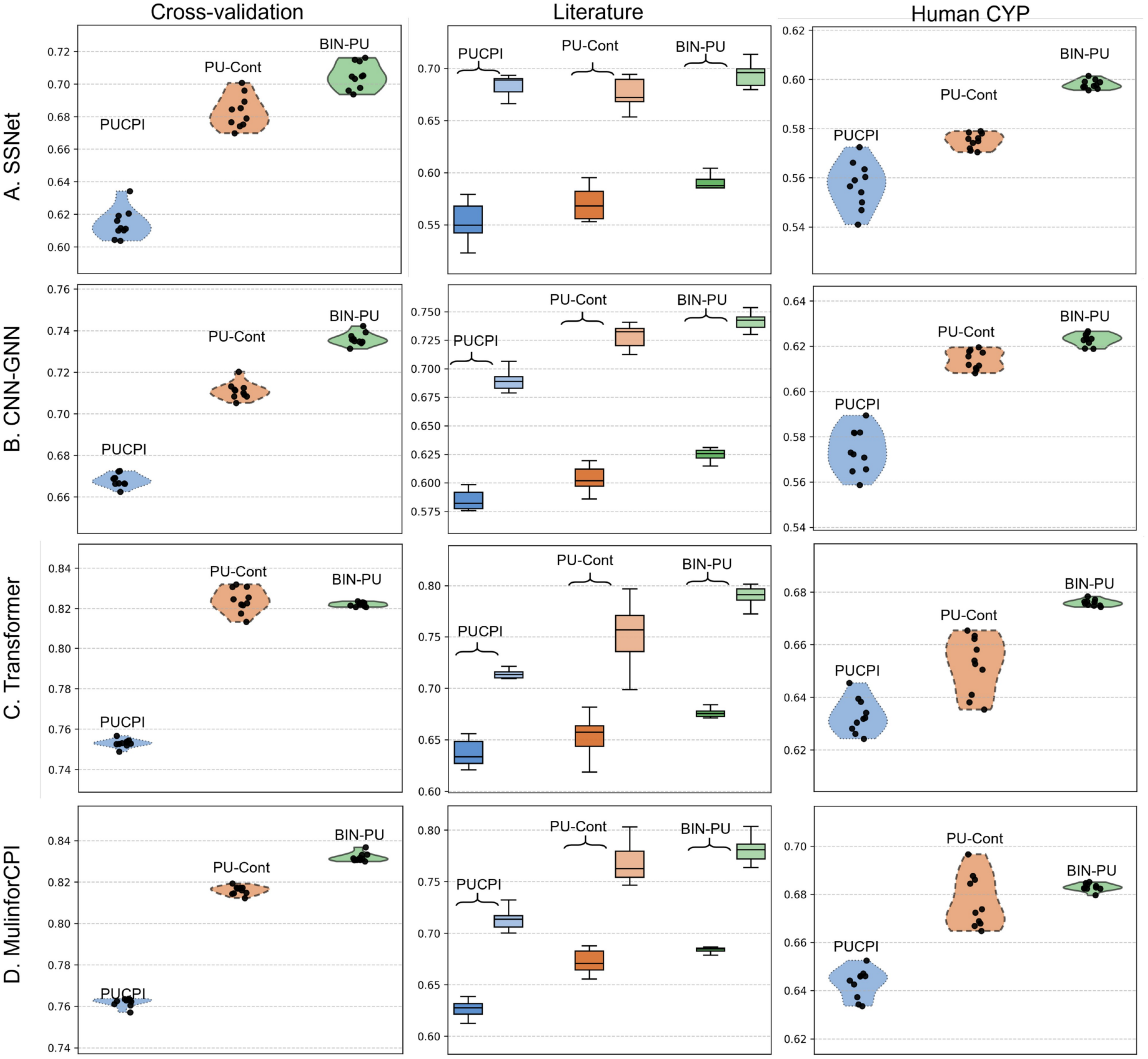
The performance comparison of BIN-PU with PUCPI and PU-Contrastive coupled with the four CPI prediction models on F1 scores. The performance is evaluated across the three experimental settings (left to right): cross-validation; external validation on bacterial literature datasets (comparing the full dataset, *N* = 129, on the left versus a subset of 41 well-studied samples on the right); and validation on the Human CYP dataset containing verified positive and negative samples with the CPI backbone model of (A) SSNet, (B)CNN-GNN, (C) Transformer, and (D) MulinforCPI.

The F1-scores of PUCPI, PU-Contrastive, and BIN-PU coupled with the CPI backbone models are illustrated in Fig. 3. BIN-PU consistently outperformed PUCPI and PU-Contrastive across all four CPI backbone models. Specifically, for the MulinforCPI backbone model, BIN-PU achieved the highest F1-score of 0.832±0.002, compared to PUCPI of 0.762±0.002 and PU-Contrastive of 0.816±0.002, reflecting a significant performance improvement of approximately 7% and 2%. BIN-PU also showed notable improvements over the other backbone models, including TransformerCPI, CNN-GNN, and SSNet. For TransformerCPI, BIN-PU obtained 0.822±0.001 compared to 0.753±0.002 (PUCPI) and 0.824±0.006 (PU-Contrastive); for CNN-GNN, BIN-PU obtained 0.736±0.003 compared to 0.668±0.003 (PUCPI) and 0.711±0.004 (PU-Contrastive); for SSNet, BIN-PU achieved 0.705±0.008 versus 0.614±0.009 (PUCPI) and 0.683±0.010 (PU-Contrastive). All improvements were statistically significant according to the Wilcoxon rank-sum test (p<0.05).

### 3.3 External validation with literature data in bacterial CYP

We evaluated the performance of BIN-PU using additional bacterial samples manually collected from the literature. We have identified bacterial CYP samples (Table 5, available as supplementary data at *Bioinformatics* online). We searched the literature published from 2000 to 2022 using the keyword, “microbial CYP family” in PubMed, limiting it to the pairs of five CYPs and 98 substrates. Finally, we collected 80 positive and 49 negative CPI samples. We confirmed that the external literature validation data were not included in the cross-validation data. For the performance assessment, we computed F1-scores using the optimal BIN-PU, PUCPI, and PU-Contrastive models, which were trained with the cross-validation data (Fig. 3B). BIN-PU showed significantly improved performance with all four CPI backbone models, compared to PUCPI and PU-Contrastive. Specifically, for MulinforCPI, BIN-PU achieved the highest F1-score of 0.684±0.004 (Wilcoxon rank-sum test *P*-value<.05) compared to PUCPI of 0.625±0.011, and PU-Contrastive of 0.672±0.010. Similarly, BIN-PU with SSNet, CNN-GNN, and TransformerCPI obtained the higher F1-scores of 0.589±0.010, 0.625±0.005, and 0.676±0.005 compared to PUCPI’s 0.553±0.018, 0.585±0.009, and 0.637±0.013 and PU-Contrastive’s 0.570±0.015, 0.604±0.011, and 0.654±0.008 (Wilcoxon rank-sum test *P*-value<.05). The overall low accuracy was mainly due to limited interaction information in the diverse substrates in the training dataset. Thus, we narrowed down the examination, focusing on 30 well-studied substrates with simple structures. When we considered the well-studied samples (N=41), the accuracy significantly improved to 79.2% by BIN-PU with TransformerCPI. This may imply the importance of comprehensive and high-quality training data for enhancing predictive performance and reliability.

### 3.4 External validation with curated human CYP data

We further evaluated the performance using human CYP data, in which negative ground truths are available, for the indirect assessment. The study was with the assumption that fundamental patterns of CYP proteins are shared across the species. We obtained the data from Integrated Protein-Ligand Interaction Database (IPLID), which comprises 17 273 unique substrates and 47 unique CYP proteins (Lim and Xie 2019). The human CYP dataset contained 25 045 positive samples and 37 277 negative samples. We applied the final models, which were trained with the cross-validation of bacteria CYP data, to the human data. Figure 3 depicts the F1-scores on the human CYP dataset. BIN-PU also outperformed PUCPI and PU-Contrastive. BIN-PU with MulinforCPI achieved the highest F1-score of 0.683±0.0015, compared to PUCPI of 0.643±0.0061 and PU-Contrastive of 0.677±0.0109 (Wilcoxon rank-sum test *P*-value<.05). Similarly, BIN-PU showed higher F1-scores than PUCPI and PU-Contrastive. The F1-scores of BIN-PU with TransformerCPI, CNN-GNN, and SSNet were 0.676±0.0012, 0.623±0.0026, and 0.598±0.0018, respectively, while PUCPI’s were 0.633±0.0065, 0.574±0.0096, and 0.557±0.0094, and PU-Contrastive’s were 0.652±0.0108, 0.614±0.0041, and 0.575±0.0031 (Wilcoxon rank-sum test *P*-value<.05).

### 3.5 Validation with uncurated bacterial CYP data

We applied the proposed models to uncurated bacterial CYP proteins with various substrates to identify their potential interactions and validated them with biological and biophysical experiments, as it is a typical practice in genome studies. Note that the previous experiments validated the models using well-established curated data only. In this experiment, we explored five CYP proteins of CYP154C9, CYP1047A9, CYP107G9, CYP106A4, and CYP106A5, which are potential candidates of novel steroid hydroxylase. For substrates, we considered four steroids of androstenedione, progesterone, nandrolone, and prednisone, known as representative substrates for CYP due to their well-established interactions with CYP. Along with the five CYP proteins and four steroids, we mainly examined their interactions focusing on: (i) the interactions of a CYP protein (CYP154C9) with the three steroids (Exp. 1–3 in Table 1), (ii) the interactions of a steroid (nandrolone) with the five CYP proteins (Exp. 3–7 in Table 1), and (iii) the interactions of a steroid (prednisone) with the two CYP proteins (Exp. 8 and 9 in Table 1).

**Figure 4:**
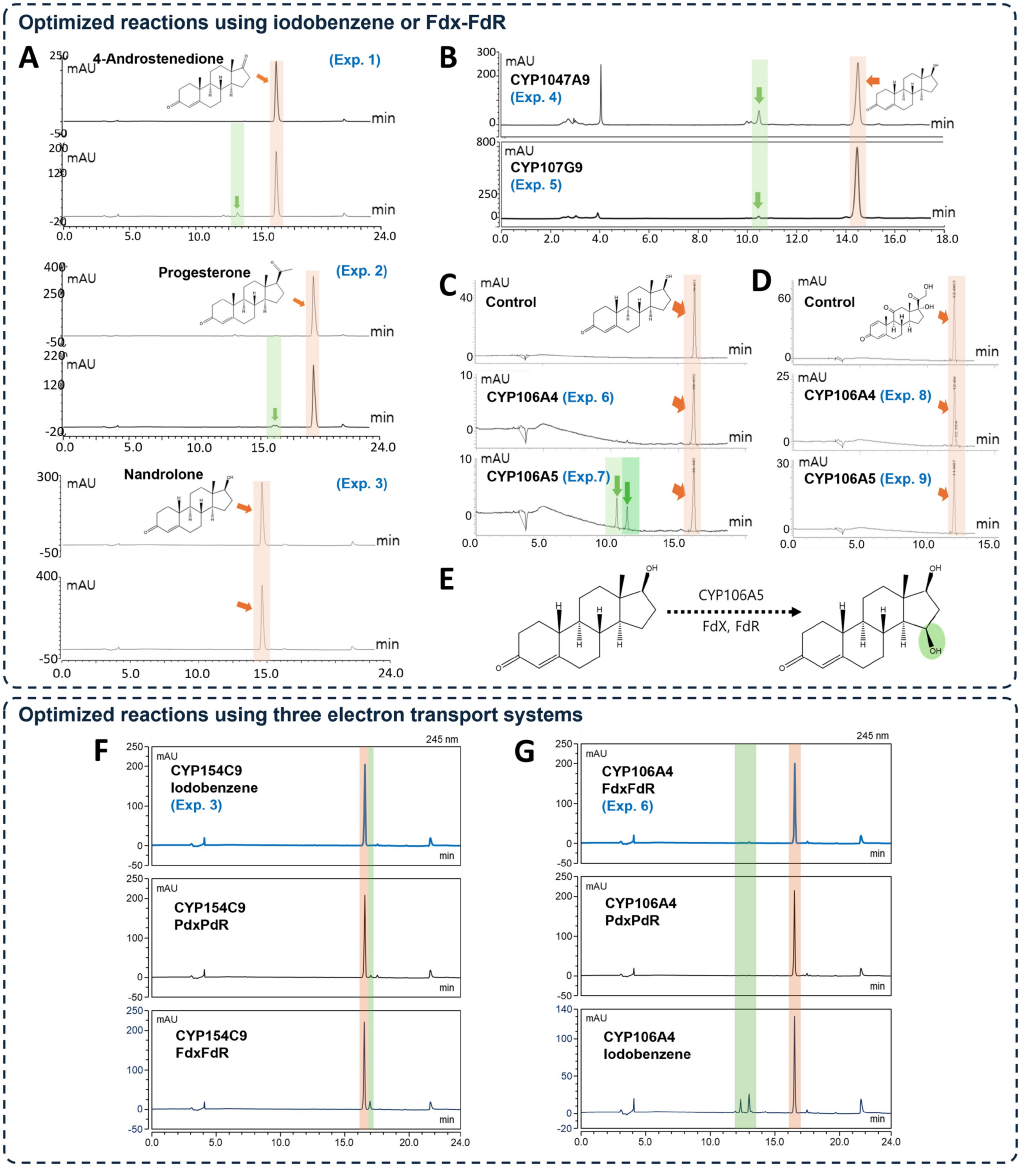
HPLC chromatogram of the *in vitro* conversion of steroid by CYPs. In (A)–(E), HPLC validations were conducted using the optimal electron transfer systems according to literature. Iodobenzene was used for CYP154C9, CYP1047A9, and CYP107G9, whereas an Fdx–FdR system was used for CYP106A4 and CYP106A5. (A) HPLC analysis of CYP154C9 with three steroids (4-androstenedione, progesterone, and nandrolone) (Exp. 1–3); (B) Activity analysis of CYP1047A9 and CYP107G9 with nandrolone (Exp. 4 and 5); (C) Comparison of nandrolone activities between CYP106A4 and CYP106A5 (Exp. 6 and 7); (D) Activity analysis of CYP106A4 and CYP106A5 with prednisone (Exp. 8 and 9). Orange arrows and boxes indicate substrate peaks, and green arrows and boxes indicate product peaks; (E) Structure of the predicted product formed by CYP106A5. In (F) and (G), additional HPLC experiments were conducted using three electron transfer systems (iodobenzene, Pdx–PdR, and Fdx–FdR) for the reactions of the substrate nandrolone with CYP154C9 and CYP106A4. (F) HPLC analysis of CYP154C9 with the three electron transfer systems; (G) Comparison of CYP106A4 activities using the three electron transfer systems. The Pdx–PdR reaction is carried out at an enzyme: Pdx:PdR ratio of 1:6:2 and is initiated by the addition of NADH. The orange box indicates the substrate peak, and the green box indicates the product peak.

We presumed their potential reactions by conducting both biological and biophysical experiments. To biologically assess the potential reactions, we cultured, purified, and conducted in vitro activity assays. We obtained bacterial strains of Streptomyces alboniger, Streptomyces sp., and Actinomycete sp. from the Korean Culture Center of Microorganisms (KCCM) and the American Type Culture Collection (ATCC) (Table 6, available as supplementary data at *Bioinformatics* online). Additionally, we obtained a Paenibacillus sp. strain isolated from Antarctica to expand the diversity of microbial sources for enzyme characterization. We identified the five CYP genes in the microorganisms based on gene sequence identity, including the conserved signature heme-binding domain [FXXGX(H/R)XCXG], and the CYP enzyme names were assigned by Dr. David Nelson (Nelson 2009). We amplified the target genes using Polymerase Chain Reaction (PCR), which were subsequently cloned into pET-28a(+) or pET-32a(+) expression vectors and expressed in recombinant *Escherichia coli* C41 (DE3) strains. The target genes were transcribed and translated into proteins, followed by purification using a Lac operon-based overexpression system and a His-tag method to obtain high-purity protein. We performed in vitro enzymatic activity assays using the purified proteins, and validated the reactions through High-Performance Liquid Chromatography (HPLC) (Note 4, available as supplementary data at *Bioinformatics* online, Fig. 4). In the HPLC analysis, inactivated CYP served as a control, allowing the detection of novel peaks corresponding to the products formed from the activity reaction. In the biological experiments, an interaction was defined as a conversion >3% by the HPLC analysis, whereas a non-interaction was defined as a conversion ≤3%. All experiments were repeated at least twice independently to ensure reproducibility, following commonly accepted guidelines for method validation in chromatographic analyses.

The HPLC analyses estimated five reactions (Exp. 1–2, 4–5, and 7) and four non-reactions (Exp. 3, 6, 8 and 9). In biological assays that use the iodobenzene electron transfer system, the HPLC analysis of CYP154C9 (peaks in green, Fig. 4A) indicated catalytic activity with androstenedione, progesterone (Exp. 1–2), while no evidence was shown for the interaction with nandrolone (Exp. 3) (Dangi *et al.* 2019). Similarly, the HPLC analysis of CYP1047A9 and CYP107G9 showed reaction with nandrolone under the same electron transfer system using iodobenzene (Fig. 4B, Exp. 4–5). In biological assays using the Fdx–FdR electron transfer system, the HPLC analysis of CYP106A4 showed no reaction with nandrolone (Exp. 6), whereas CYP106A5 showed reactions with nandrolone (Exp. 7), although they have approximately 55% sequence similarity (Fig. 4C) (Kim *et al.* 2017). In contrast, CYP106A4 and CYP106A5 showed no catalytic activity with prednisone (Fig. 4D, Exp. 8–9) under Fdx–FdR electron transfer system. Notably, HPLC of CYP106A5 revealed two distinct product peaks, with one matching the retention time of a 15-beta-hydroxylated nandrolone reported for BaCYP106A2 (Fig. 4E, Fig. 2, available as supplementary data at *Bioinformatics* online) (Kim *et al.* 2017). Overall, the novel CYPs used in this study exhibited relatively low expression levels and weak catalytic activities, which could be enhanced through further optimization of the experimental conditions. Because biological enzymatic experiments are highly sensitive to both biochemical factors (e.g. substrate hydrophobicity, electron donor systems) and environmental conditions (e.g. temperature, pH, and buffer composition), all experiments were conducted under the conditions previously reported to be optimal for each CYP.

To further evaluate the catalytic potential of CYP154C9 and CYP106A4, we conducted additional *in vitro* experiments using alternative electron transfer systems (Table 2 and Fig. 4F and G). The *in vitro* reactions were performed using nandrolone as an interesting substrate and three electron transport systems for CYP154C9 and CYP106A4. These systems included iodobenzene and Fdx-FdR, which had previously been tested for activity with CYP154C9 and CYP106A4, respectively (Fig. 4F and G). Additionally, putidaredoxin–putidaredoxin reductase (Pdx–PdR), previously shown to react with CYP154C isoforms, was also included (Subedi *et al.* 2021, Paudel *et al.* 2024). Consequently, in vitro reactions confirmed that CYP154C9 reacted with Fdx-FdR to produce a product, while CYP106A4 converted iodobenzene into two products (Table 2 and Fig. 4F and G). This reaction has not been reported for either CYP, and in particular, products with lower polarity than the substrate were rarely observed in the CYP154C family (Dangi and Oh 2019).

Then, we conducted biophysical experiments using homology modeling and molecular docking to further assess potential protein–ligand interactions. As the 3D structures of the five CYP strains were not available in the Protein Data Bank, homology models of the CYP proteins were generated using the Chai-1 server (Boitreaud *et al.* 2024). The substrate compounds were retrieved from the PubChem database. Binding affinity scores and distances between the heme group and the nearest carbon atom by AutoDock Vina (version 1.2.3) and chimeraX, and the protein–compound interactions were measured by using the MOE software (the details are in Note 5, available as supplementary data at *Bioinformatics* online and Table 7, available as supplementary data at *Bioinformatics* online) (Meng *et al.* 2023). The results of this analysis are presented in Table 1.

In biophysical experiments, substrate affinities were evaluated by molecular docking, with a positive result indicating a possible docking pose. On the other hand, if the distance between the heme iron and the substrate carbon atom exceeded 5 Å, the result was classified as negative. This does not necessarily imply that interactions are completely impossible. The biophysical assessments revealed five strong binding affinity scores in Exp. 2–6 and comparatively low affinity scores in Exp. 1 and 7–9 (Table 1). Binding energies in molecular docking are typically expressed as negative values, where lower (more negative) scores reflect stronger binding affinities. In Exp. 1–3, CYP154C9 was docked with three substrates, and the docked poses indicated potential reactions at carbon 2. It exhibited strong binding scores of −7.19 kcal/mol with progesterone in Exp. 2 and −8.27 kcal/mol with nandrolone in Exp. 3, whereas a low affinity of −6.35 kcal/mol was observed with 4-androstenedione in Exp. 1. This observation aligns with the known catalytic function of CYP154C2, another member of the CYP154C family, which has been reported to catalyze hydroxylation at the same carbon position 2α (Gao *et al.* 2023). In Exp. 4–5, CYP1047A9 and CYP107G9 showed strong binding affinities of −8.24 and −7.66 kcal/mol, respectively, implying possible reactivity at carbon 2. However, these novel CYPs remain uncharacterized due to limited research on similar family members. In Exp. 6–9, docking analysis of CYP106A4 and CYP106A5 revealed two substrate binding poses. The interaction between CYP106A4 and nandrolone in Exp. 6 exhibited the strongest affinity (−8.48 kcal/mol), suggesting potential hydroxylation at carbon 15. This is supported by studies on CYP106A2, CYP106A6, and other CYP106A family members known to catalyze 15β-hydroxylation of steroid substrates (Schmitz *et al.* 2018, Kim *et al.* 2023). Comparatively low binding energies were observed in Exp. 7–9 (−6.61, −6.31, and −6.15 kcal/mol, respectively). The low affinity observed in prednisone interactions (Exp. 8–9) may be due to steric hindrance from the complex branching at position C17, impeding effective binding to the active site.

**Figure 5:**
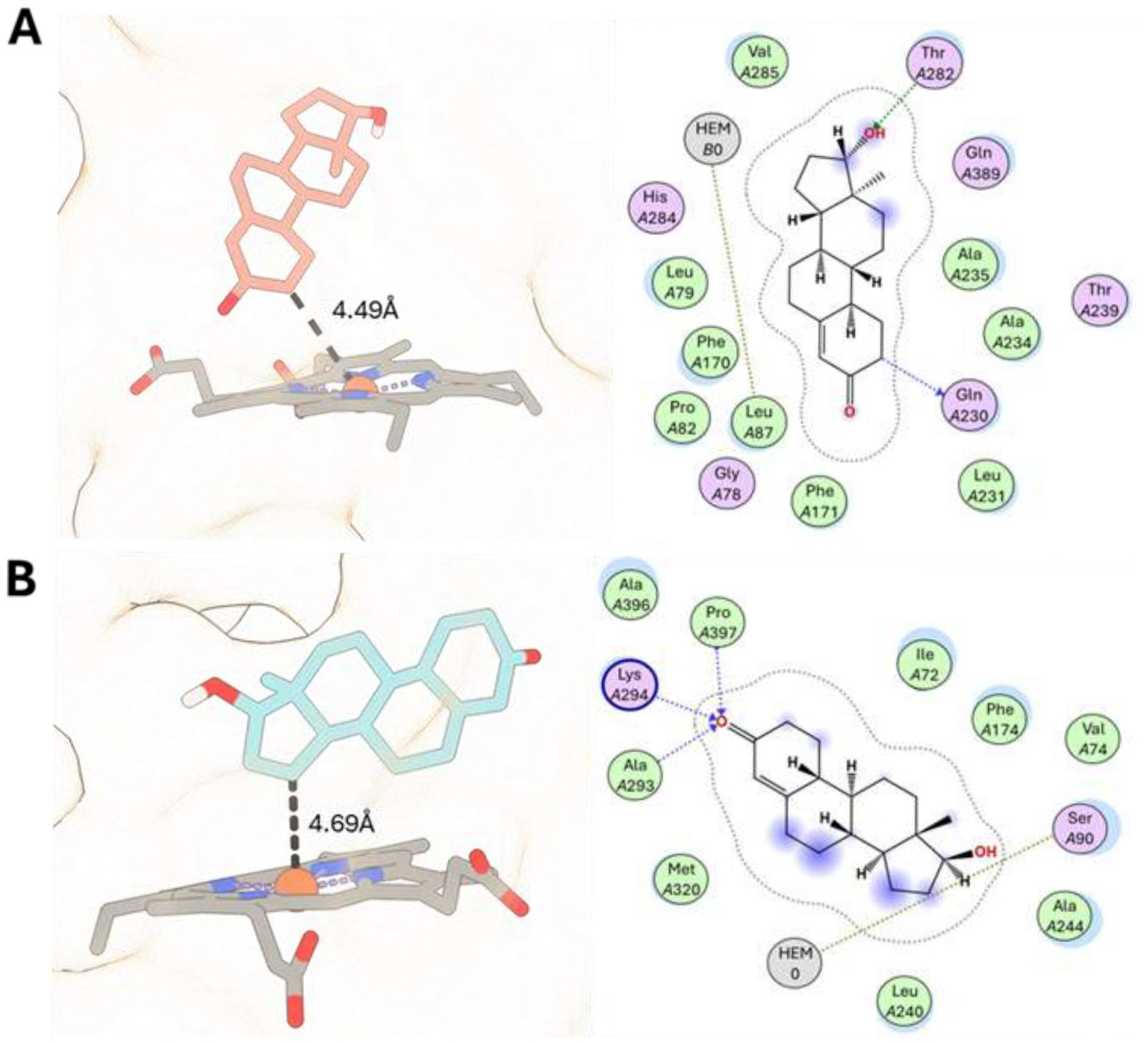
Binding mode in active site and 2D molecular interaction diagrams of the best docked compounds. (A) Active site of CYP154C9 with nandrolone. (B) Binding pose of CYP106A4 protein with nandrolone and their interaction.

Finally, based on the biological and biophysical assessment, we presumably concluded seven interactions in Exp. 1–7, and two non-interactions in Exp. 8 and 9. The product formation in Exp. 7, which was confirmed by the biological experiment, suggests the possibility of a novel product hydroxylated at carbon 16, as supported by the biophysical analysis. In contrast, Exp. 3 and 6, which performed electron transport system optimization experiments, were further analyzed using MOE to investigate interactions between amino acids for more detailed insights. Further analysis of the docking interactions revealed potential binding patterns for each steroid compound (Fig. 5 and Fig. 3, available as supplementary data at *Bioinformatics* online). Exp. 6 exhibited the strongest binding affinity by AutoDock Vina among the nine experiments, where the ligand formed a backbone acceptor hydrogen bond with amino acid residues Ala293, Lys294, and Pro397. Exp. 3, showing the second strongest binding affinity by AutoDock Vina, observed a hydrogen bond with the residue Thr282. These docking results offered structural insights of the binding modes and potential reactivity of the steroid compounds with the tested five CYP enzymes (Fig. 3, available as supplementary data at *Bioinformatics* online).

CYPs comprise numerous isoenzymes with diverse active sites and chemical properties, resulting in broad substrate selectivity and making structure-based predictions challenging (Wei *et al.* 2024). Docking simulation, a computational approach for designing protein–ligand interactions, computes binding free-energy estimates and applies scoring functions to evaluate likely possible interactions (Oostenbrink 2014). However, the structural diversity of CYP isoforms can generate false-positive results, especially when scoring functions overestimate compatibility in sterically restricted or chemically unsuitable environments (Khanjiwala *et al.* 2019). To address these challenges, we performed structural comparisons of CYP154C9 and CYP106A4 with well-characterized enzymes (CYP154C4, CYP106A2) that exhibit high reactivity and substrate specificity (Janocha *et al.* 2016, Kim *et al.* 2017, Dangi *et al.* 2019). Due to the unavailability of crystal structures for CYP154C9 and CYP106A4, homology modeling was used to generate their structures, and later aligned with the crystal structures of CYP154C4 (PDB 6A7J) and CYP106A2 to examine differences in active-site geometry and substrate accessibility. These comparisons revealed that both CYP154C9 and CYP106A4 possess substrate-access channels that are notably narrower than those of their respective isoforms (Fig. 4, available as supplementary data at *Bioinformatics* online). In CYP154C4 (Fig. 4A, available as supplementary data at *Bioinformatics* online), a tunnel-like cavity maintained by an extended methionine residue provides an open path for substrate entry and product release, whereas in CYP154C9, the corresponding leucine residue rotates inward, constricting the channel and reducing cavity volume. Electrostatic surface analysis further highlights this distinction: CYP154C4 exhibits a deep, electronegative tunnel, while CYP154C9 presents a shallower, mixed-charge pocket shaped by the occluding leucine side chain. A similar pattern was observed in the CYP106 family. CYP106A2 contains a deep, well-defined cavity that can accommodate a diverse range of steroid substrates. Similarly, CYP106A4 features a slightly shallower pocket influenced by a unique loop insertion that alters the local surface topology and may restrict substrate accessibility and orientation (Fig. 4B and C, available as supplementary data at *Bioinformatics* online). These comparisons explain why CYP154C9 and CYP106A4 did not yield detectable reaction products despite favorable docking scores. Overall, the structural analysis aligns with the low conversion rates observed in biological assays and clarifies the discrepancies between docking predictions and experimental outcomes.

We applied PUCPI, PU-Contrastive, and BIN-PU to predict the potential interactions between the CYPs and steroids (Tables 8 and 9, available as supplementary data at *Bioinformatics* online, and Table 1). For the evaluation, we mainly considered BIN-PU with TransformerCPI and MulinforCPI, which produced the best models in the previous experiments, while the predictive results with other models are also shown. BIN-PU with TransformerCPI and MulinforCPI identified seven potential interactions in Exp. 1–7 and two non-interactions in Exp. 8–9 (Table 1). Specifically, in the positive predictions in Exp. 1–7, the highest prediction score was observed in Exp. 1 for the CYP154C9-androstenedione reaction, with a predictive score of 0.941, followed by Exp. 2 (0.839) and Exp. 7 (0.717) using BIN-PU with TransformerCPI. For BIN-PU with MulinforCPI, higher prediction scores were observed in Exp. 4 (0.584) and Exp. 5 (0.880). On the other hand, Exp. 3 and 6 showed low predictive scores of 0.530 and 0.490, respectively, by BIN-PU with TransformerCPI. In Exp. 8 and 9, BIN-PU with TransformerCPI and MulinforCPI predicted no interaction, with scores of (0.117 and 0.140), and (0.255 and 0.330), respectively.

## 4 Conclusions

We introduced a novel positive-unlabeled learning strategy (BIN-PU) for CPI prediction, especially where curated negative samples are not available. BIN-PU integrates unlabeled samples with truly positive samples, mitigating the challenge of lack of negative samples. We conducted intensive experiments with various experimental settings and bacterial CYP datasets, and the experiments demonstrated the effectiveness of our approach across various CPI backbone architectures with a significantly improved predictive performance than PUCPI and PU-Contrastive. We also applied it to uncurated CYP and substrates, and the potential reaction predictions were validated with biological and biophysical experiments.

Although BIN-PU was applied and assessed using only bacterial CYPs in this study, it could be extended to other bacterial proteins to identify bacterial protein–compound interactions. However, the generalization should consider the biological diversity inherent to bacterial protein families. In particular, bacterial protein families have entirely different domain architectures and biochemical functions. For instance, bacterial CYPs include conserved heme-binding motif, substrate-recognition regions, and monooxygenase activity, whereas bacterial kinases have ATP-binding pockets and phosphorylation-specific motifs (e.g. Ser/Thr-Pro or Ser-X-X-X-Ser-Pro) that are structurally and functionally distinct from those in CYPs (Cheng *et al.* 2014, Blum *et al.* 2025). Such domain- and functional-level differences may introduce generalization gaps. Nevertheless, BIN-PU captures physicochemical interaction patterns rather than motif-specific features only, providing a basis for extending the framework to additional bacterial proteins when sufficient positive-only data are available. Expanding the training dataset to include a broader range of bacterial protein families may improve the model’s generalization in bacterial protein–compound interaction studies.

In this study, we considered both activators and inhibitors as reactions due to the limited availability of labeled data of inhibitors and activators for the interactions in this study. Nevertheless, CPI prediction using BIN-PU can be an effective strategy in identifying potential substrates in early-stage biocatalytic applications or virtual screening processes. The limitation could be further tackled by integrating additional biochemical data or training with labeled datasets of activators and inhibitors available in the future.

We empirically optimized the biological assay conditions specific to each CYP enzyme. However, experimental outcomes in CYP systems are highly sensitive to biochemical conditions, including electron transfer partners, substrate properties, and reaction environments that may impact the detection of catalytic activity. These variables can result in low or undetectable product formation. In particular, the reactions of CYP154C9 (Exp. 3) and CYP106A4 (Exp. 6) with nandrolone in Section 3.5 initially showed no catalytic activity in HPLC assays, despite favorable docking scores in both biophysical and computational predictions. However, when we incorporated alternative electron transfer systems, it detected product formation, demonstrating that the interactions correspond to catalytically feasible reactions under appropriate biochemical conditions. These experiments demonstrate the complexity of biologically validating protein–compound interactions through wet-lab assays and indicate that computational approaches, such as BIN-PU, can serve as an additional line of evidence in cases where biochemical assays are limited by sensitivity or variability.

We demonstrates potential deep learning-based applications using only positive interaction labels by mainly assessing it with bacterial CYP proteins and related substrates. Future work for diverse ranges of bacterial proteins and substrates will be required to generally apply it for bacteria protein-substrate interactions prediction. Eventually, prediction of novel bacterial enzyme-substrate interactions may serve as a foundation for pharmaceutical applications and enhance biocatalysis and drug discovery.

## Supplementary Material

btag067_Supplementary_Data

## Data Availability

The data and the source code are available in the Zenodo repository at https://doi.org/10.5281/zenodo.18023528. The code used to develop the model, perform the analyses, and generate all results in this study is publicly available at GitHub (https://github.com/datax-lab/cyp) under the MIT license.
