# Supplementary material for: Prediction of bacterial protein–compound interactions with only positive samples
Source: Bioinformatics. 2026 Feb 18;42(3):btag067. doi: 10.1093/bioinformatics/btag067 (PMC12975285; doi:10.1093/bioinformatics/btag067)
Supplement: btag067_Supplementary_Data [file btag067_supplementary_data.pdf]

Supplementary: Prediction of bacterial protein-compound interactions with only positive samples

Ki-Hwa Kim[1†], Avinash Yaganapu[2†], Sai Kosaraju[3], Aashish Bhatt[4], Yun Lyna Luo[4], Sai Phani Parsa[2], Juyeon Park[5], Hyun Lee[5], Jun Hyuck Lee[6], Tae-Jin Oh[1,7,8*] and Mingon Kang[2*]

[1] Genome-based BioIT Convergence Institute, Asan, Republic of Korea

[2] Department of Computer Science, University of Nevada, Las Vegas, Las Vegas, NV, USA

[3] Department of Computer Science, California State Polytechnic University, Pomona, CA, USA

[4] Department of Biotechnology and Pharmaceutical Sciences, Western University of Health Sciences, Pomona, California, USA

[5] Department of Computer Science, Sun Moon University, Asan, Republic of Korea

[6] Division of Life Sciences, Korea Polar Research Institute, Incheon, Republic of Korea

[7] Department of Life Science and Biochemical Engineering, Sun Moon University, Asan, Republic of Korea

[8] Department of Pharmaceutical Engineering and Biotechnology, Sun Moon University, Asan, Republic of Korea

[†]The first authors: Kihwa Kim and Avinash Yaganapu share the co-first authorships
*Corresponding authors: Tae-Jin Oh and Mingon Kang

Supplementary Note S1. Dataset gathering criteria for model development

We defined the criteria for data inclusion and exclusion for the data gathering. Keywords for inclusion included "LANOSTEROL 14-ALPHA-DEMETHYLASE", "P450 MONOOXYGENASE", and "P450 epoxidase" to capture relevant enzymatic activities and interactions. In contrast, exclusion keywords included "cyclophilin" and "Pyruvate dehydrogenase" to avoid non-relevant proteins, ensuring focus on true CYP interactions. The complete list of keywords is in Table S1. Additionally, we analyzed ligand characteristics, including carbon number and functional type, to filter out inactive compounds or those located outside the active site. We excluded ligands marked as buffers, cofactors, or irrelevant small molecules (e.g., DMSO or buffer ions). This data refinement is shown in Table S2, which categorizes ligands by their inclusion criteria.

Table S1. Protein names that are included or excluded based on keyword search results in the PDB database.

	Keywords
Keywords for inclusion	“LANOSTEROL 14-ALPHA-DEMETHYLASE”, “P450 MONOOXYGENASE”, “PROSTACYCLIN SYNTHASE”, ”P450 epoxidase”, “STEROL 14ALPHA-DEMETHYLASE”, ”P450cin”, “ALLENE OXIDE SYNTHASE”, “6-DEOXYERYTHRONOLIDE B HYDROXYLASE”, “PROSTAGLANDIN I SYNTHASE”, “putative cytochrome P450”, “STEROL 14-ALPHA-DEMETHYLASE”, “CAMPHOR 5-MONOOXYGENASE”, “LANOSTEROL 14-ALPHA DEMETHYLASE”, “PIMD PROTEIN”, “STEROL 14 ALPHA-DEMETHYLASE”, ”P-450-LIKE PROTEIN”, “1,5-DIHYDROXYVITAMIN D(3) 4-HYDROXYLASE”, “P450 HEME-THIOLATE PROTEIN”, “STEROL 14-ALPHA DEMETHYLASE”, ”Vitamin D hydroxylase”, “Cholesterol 24-hydroxylase”, “Fatty acid alpha-hydroxylase”, “CHOLESTEROL 4-HYDROXYLASE”, “PUTATIVE MONOOXYGENASE”, “CHOLESTEROL SIDE-CHAIN CLEAVAGE ENZYME”, “TERMINAL OLEFIN-FORMING FATTY ACID DECARBOXYLASE”, “STEROID 1-HYDROXYLASE”, “P450-like protein”, “STEROID 17-ALPHA-HYDROXYLASE/17,0 LYASE”, “PENTALENIC ACID SYNTHASE”, “CHOLESTEROL 7-ALPHA-MONOOXYGENASE”, “Cytochrome P-450”, “CYP17A1”, “MYCOCYCLOSIN SYNTHASE”, “STEROL 14-DEMETHYLASE”, “PENTALENOLACTONE SYNTHASE”, “AROMATASE”, “P450-LIKE ENZYME”, “FERRUGINOL SYNTHASE”, “Fatty-acid peroxygenase”, “PROTEIN LUTEIN DEFICIENT 5”, “METHYL-BRANCHED LIPID OMEGA-HYDROXYLASE”, “HYPOTHETICAL CYTOCHROME P450”, “Aromatic O-demethylase”, “CYTOCHROME P450 BM-3”, “SalCYP”
Keywords for exclusion	“cyclophilin”, “CYTOCHROME P460”, “GREEN FLUORESCENT PROTEIN”, “Pyruvate dehydrogenase”, “NUCLEAR RECEPTOR SUBFAMILY 5 GROUP A MEMBER”, “ferredoxin”, “Histone H3.1”, “AA3-600 quinol oxidase subunit I”, “CYTOCHROME-C55”, “HEMOGLOBIN”

Table S2. Ligand characteristics used for dataset refinement.

Ligand ID	Carbon number	Characteristic
TRS	C4	No substrate
SRT	C4	No substrate
MLA	C3	No substrate
GOL	C3	Buffer
SPK	C10	No substrate
MES	C6	Buffer
PEG	C4	Buffer
XE	C0	No substrate
BME	C2	No substrate
DTT	C4	No substrate
IPA	C3	No substrate
DMS	C2	Solvent
BEN	C7	No substrate
SIN	C4	Buffer
IMD	C3	Buffer
PG0	C5	Buffer
MRD	C6	Buffer
EPE	C8	Buffer
MPD	C6	No substrate
TLA	C4	No substrate
FMT	C1	Small molecule
PGE	C6	No substrate
MTN	C10	Solvent
PG4	C8	Buffer
1T4	C18	No substrate
MSE	C5	Linker
CSD	C3	Linker
CME	C5	Linker
CSO	C3	Linker
1PE	C10	Located outside the active site
5KK	C18	No substrate
SPD	C7	No substrate
RU8	C34	Photosensitizer
TAM	C7	No substrate
DIO	C4	No substrate
BCN	C6	Located outside the active site
MPO	C7	Buffer

SER	C3	Located outside the active site
SPM	C10	Located outside the active site
17Q	C19	Located outside the active site
OXY	C0	Small molecule
HOA	C0	Small molecule
NO	C0	Small molecule
CMO	C1	Small molecule
FMN	C17	Cofactor
FES	C0	Cofactor
MNR	C34	Cofactor
HTG	C13	Cofactor
1N0	C14	Cofactor

Supplementary Note S2. Model implementation.

We implemented the four CPI backbone models, including CNN-GNN, TransformerCPI, SSNet, and MulinforCPI. For the preprocessing, we followed their proposed encoding schemes for proteins and compounds. Specifically, for CNN-GNN, we encoded protein sequences using overlapping n-grams (3-mers). Each n-gram was converted into a trainable embedding vector of dimension 128, and the embedding was processed through stacked convolutional layers, followed by max-pooling to extract local sequence motifs. For compound encoding, we converted SMILES strings into molecular graphs using RDKit. Each atom was encoded as a feature vector that included atom type, hybridization, aromaticity, formal charge, degree, and number of hydrogens, while bonds were encoded in an adjacency matrix specifying bond types. These molecular graphs were processed using a graph neural network to generate compound-level graph embeddings. For TransformerCPI, we trained a Word2Vec model on the protein sequences from the training dataset to learn task-specific embeddings for amino acids. Each amino acid was mapped to its corresponding dense vector, and the sequence of embeddings was processed through transformer encoder layers. For compound encoding, we computed 34-dimensional molecular descriptors for each compound using RDKit. These descriptors summarize atom types, hybridization, valence, aromaticity, and bond connectivity. We fed the descriptor vectors into a feedforward encoder and processed them together with the protein embeddings using attention layers. For SSNet, we computed structural encodings from protein 3D conformations. We extracted C-α atom coordinates from the PDB files, and curvature (κ) and torsion (τ) values were calculated for each residue to generate a two-channel (2×sequence-length) representation. We processed these κ–τ sequences using multi-branch one-dimensional convolutional neural networks with kernel sizes of 5, 10, 15, and 20, followed by global max-pooling. For compound encoding, we constructed molecular graphs from SMILES strings using RDKit, following the same atom and bond feature extraction procedure as done in CNN-GNN. We additionally computed molecular fingerprints, including ECFP-like circular fingerprints. We concatenated molecular fingerprints with the graph-derived features to form hybrid dense vectors, which we passed through fully connected layers to generate compound embeddings compatible with the protein κ–τ representations. For MulinforCPI, we computed protein embeddings using a pre-trained protein language model (ESM), which generates contextualized residue-level representations that capture evolutionary and semantic characteristics of the sequence. Each residue was mapped to a 1280-dimensional embedding vector generated by the ESM model, and the resulting sequence embeddings were provided as input for the multi-level interaction encoder of MulinforCPI. For compound encoding, we converted SMILES strings into molecular graphs using RDKit. Each atom was represented by a feature vector consisting of atom type, atomic number, valence, hybridization, aromaticity, formal charge, and number of hydrogens. Bond connectivity was encoded using an adjacency matrix with bond-type annotations. These molecular graphs were processed with GIN-based graph neural networks to produce atom-level and graph-level embeddings. We combined the ESM protein embeddings and the GIN compound embeddings through a multi-level cross-attention mechanism, enabling residue–atom and global interaction modeling.

For BIN-PU, we followed the procedure illustrated in Fig. 2. We first generated unlabeled data by creating pairwise combinations of proteins and compounds, excluding known positive interactions. We randomly divided the unlabeled data into K subsets (bins) and trained multiple CPI classifiers, each using a combination of truly positive samples and one subset of unlabeled samples for each bin. We aggregated the posterior probabilities from these

classifiers to assign pseudo labels, selecting high-confidence samples as pseudo positives and pseudo negatives. We then trained the CPI backbone models (CNN-GNN, TransformerCPI, SSNet, and MulinforCPI) in a fully supervised manner using truly positive, pseudo positive, and pseudo negative samples together. During training, we applied a weighted positive loss function to give higher importance to truly positive samples and to minimize misclassification of biologically relevant interactions.

For PUCPI, we implemented the training as described in the original study. All unlabeled samples were treated as negatives. We utilized the same datasets, data splits, and model configurations as in BIN-PU to ensure a fair comparison. We trained PUCPI coupled with the CPI backbone models directly with truly positive and unlabeled (assumed negative) samples. PUCPI did not include any pseudo-label generation, however, we used the pseudo-labels generated by BIN-PU for evaluating the performance of PUCPI. We also considered the original PUCPI using a biased-SVM without experimentally validated negative samples. PUCPI was applied to human enzyme data by utilizing substructures and the PFAM database for data preprocessing. However, due to the unavailability of the database for bacterial CYP data, we slightly modified the training setting of PUCPI. We performed the same data preprocessing of CNN-GNN for PUCPI. Specifically, we first extracted feature vectors for protein and compound vectors. The compound vector was multiplied with an adjacency vector to include bond information. We fed the multiplied vector to a 1D CNN to match the number of output channels as a protein vector. We computed the tensor product between the protein vector and the converted compound vector. Following that, a biased-SVM classifier was trained on the tensor product by providing more weight to known positives. The hyperparameters, including c (tradeoff between training error and margin) and j (cost factor) of biased-SVM loss function, were tuned using the validation data. PUCPI coupled with biased-SVM achieved the F1 scores of 0.673 ± 0.017 (cross-validation), 0.571 ± 0.001 (human CYP), and 0.540 ± 0.003 (literature data). We used the same training, validation, and test data for a fair comparison.

For PU-Contrastive, we implemented the contrastive PU-learning methodology as described in the original study. We generated two stochastic augmentations for each positive sample, and the InfoNCE contrastive loss was employed to maximize the similarity between the augmented views of positive samples while minimizing the similarity to unlabeled samples. PU-Contrastive did not include any pseudo-label generation, however, we used the pseudo-labels generated by BIN-PU for evaluating the performance of PU-Contrastive. The hyperparameters, including the temperature parameter (τ) in the contrastive loss, the embedding dimension, the batch size, and the optimizer (i.e., Adam with learning rate 1e-3), were tuned using the validation data.

Supplementary Note S3. Hyperparameter tuning for BIN-PU.

We obtained optimal hyperparameters, including the bin size and the lambda value for the weighted positive loss function, that optimized the performance on the validation data for each experiment. Specifically, the optimal number of bins (bin size) was obtained to maximize the Spies Capture Rate (SCR). We evaluated bin sizes of 10, 20, and 30 across the three CPI backbone models and selected 20 as the optimal bin size, as it consistently achieved the highest SCR (Table S3, Fig. S1A).

We performed an ablation study to examine the sensitivity of the thresholds (θ and λ) that determine pseudo-label on BIN-PU. We varied the top-*k* percentile threshold for determining pseudo-positive and pseudo-negative samples from top-10% to top-50% across all CPI backbones (CNN-GNN, TransformerCPI, SSNet, and MulinforCPI). BIN-PU achieved the highest predictive performance (i.e., F1-scores) across all threshold settings, with the top-20% threshold resulting in the most consistent outcomes across all CPI backbones. The complete results of this analysis are provided in Table S4.

We also tuned the lambda parameter in the weighted positive loss function to control the weight assigned to truly positive samples. We tested lambda values of 1, 10, 100, and 1000 across the four CPI backbone models and selected lambda = 100 as the optimal value, as it consistently resulted in the highest F1-scores across experiments (Table S4, Fig. S1B).

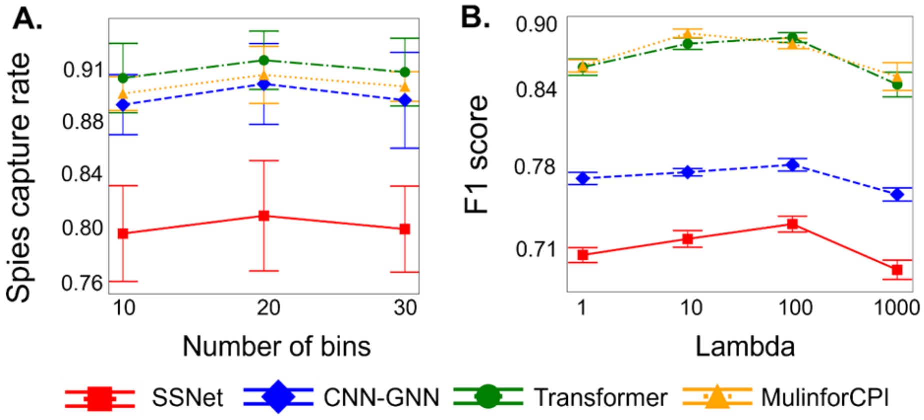

Figure S1. Comparison of BIN-PU performance across varying parameters using error bar plots. (A) Spies capture rate across multiple bins, (B) Lambda values vs F1 score during training of CPI model with weighted positive loss function, and the error bars represent the standard deviation across 10 iterations for each model.

Table S3. Performance comparison varying bin sizes.

Model	10 bins	20 bins	30 bins
CNN-GNN	0.891 ± 0.00031	**0.904 ± 0.001664**	0.886 ± 0.00142
TransformerCPI	0.908 ± 0.00224	**0.920 ± 0.00144**	0.914 ± 0.00096
SSNet	0.81 ± 0.0052	**0.846 ± 0.0064**	0.832 ± 0.00664
MulinforCPI	0.895 ± 0.0031	**0.914 ± 0.0021**	0.902 ± 0.001

Table S4. Performance comparison of CPI backbone model with varying lambdas.

Model	λ	Top-10%	Top-20%	Top-30%	Top-40%	Top-50%
CNN-GNN	1	0.771 ± 0.0032	0.766 ± 0.0033	0.682 ± 0.0041	0.660 ± 0.0044	0.612 ± 0.0047
	10	0.759 ± 0.0036	0.770 ± 0.0056	0.687 ± 0.0043	0.660 ± 0.0046	0.616 ± 0.0049
	100	0.763 ± 0.0031	**0.774 ± 0.0017**	0.692 ± 0.0031	0.664 ± 0.0036	0.624 ± 0.0041
	1000	0.748 ± 0.0031	0.752 ± 0.0011	0.670 ± 0.0030	0.643 ± 0.0033	0.600 ± 0.0040
TransformerCPI	1	0.852 ± 0.0060	0.854 ± 0.0078	0.770 ± 0.0064	0.740 ± 0.0068	0.700 ± 0.0073
	10	0.881 ± 0.0015	0.872 ± 0.0006	0.789 ± 0.0019	0.760 ± 0.0022	0.720 ± 0.0027
	100	0.873 ± 0.0015	**0.876 ± 0.0018**	0.791 ± 0.0020	0.760 ± 0.0025	0.718 ± 0.0029
	1000	0.834 ± 0.0019	0.838 ± 0.0007	0.752 ± 0.0018	0.721 ± 0.0022	0.678 ± 0.0027
SSNet	1	0.708 ± 0.0028	0.704 ± 0.0015	0.622 ± 0.0034	0.598 ± 0.0037	0.556 ± 0.0040
	10	0.715 ± 0.0028	0.718 ± 0.0034	0.634 ± 0.0037	0.609 ± 0.0039	0.565 ± 0.0043
	100	**0.733 ± 0.0039**	0.728 ± 0.0033	0.646 ± 0.0038	0.619 ± 0.0042	0.575 ± 0.0045
	1000	0.681 ± 0.0031	0.686 ± 0.0089	0.602 ± 0.0040	0.575 ± 0.0043	0.540 ± 0.0048
MulinforCPI	1	0.868 ± 0.0018	0.864 ± 0.0075	0.780 ± 0.0021	0.754 ± 0.0025	0.710 ± 0.0030

**Model**	**λ**	**Top-10%**	**Top-20%**	**Top-30%**	**Top-40%**	**Top-50%**
	10	0.884 ± 0.0016	0.882 ± 0.0006	0.799 ± 0.0019	0.772 ± 0.0023	0.725 ± 0.0028
	100	0.872 ± 0.0014	**0.886 ± 0.0017**	0.805 ± 0.0020	0.776 ± 0.0025	0.728 ± 0.0030
	1000	0.839 ± 0.0019	0.848 ± 0.0007	0.763 ± 0.0023	0.734 ± 0.0027	0.688 ± 0.0032

Table S5. List of CYPs included in literature papers.

CYP name	PDB ID	Reference
*Ba*CYP106A2	5XNT	Kim, K. H., Lee, C. W., Dangi, B., Park, S. H., Park, H., Oh, T. J., & Lee, J. H. (2017). Crystal structure and functional characterization of a cytochrome P450 (BaCYP106A2) from Bacillus sp. PAMC 23377. Journal of Microbiology and Biotechnology, 27(8), 1472-1482.
CYP106A2	4YT3	Schmitz, D., Janocha, S., Kiss, F. M., & Bernhardt, R. (2018). CYP106A2—a versatile biocatalyst with high potential for biotechnological production of selectively hydroxylated steroid and terpenoid compounds. Biochimica et Biophysica Acta (BBA)-Proteins and Proteomics, 1866(1), 11-22.
CYP109E1	5L91	1. Jóźwik, I. K., Kiss, F. M., Gricman, Ł., Abdulmughni, A., Brill, E., Zapp, J., ... & Thunnissen, A. M. W. (2016). Structural basis of steroid binding and oxidation by the cytochrome P450 CYP 109E1 from Bacillus megaterium. The FEBS Journal, 283(22), 4128-4148.<br>2. Putkaradze, N., Litzenburger, M., Hutter, M. C., & Bernhardt, R. (2019). CYP109E1 from Bacillus megaterium Acts as a 24-and 25-Hydroxylase for Cholesterol. ChemBioChem, 20(5), 655-658.<br>3. Abdulmughni, A., Jóźwik, I. K., Putkaradze, N., Brill, E., Zapp, J., Thunnissen, A. M. W., ... & Bernhardt, R. (2017). Characterization of cytochrome P450 CYP109E1 from Bacillus megaterium as a novel vitamin D3 hydroxylase. Journal of Biotechnology, 243, 38-47.<br>4. Putkaradze, N., König, L., Kattner, L., Hutter, M. C., & Bernhardt, R. (2020). Highly regio- and stereoselective hydroxylation of vitamin D2 by CYP109E1. Biochemical and biophysical research communications, 524(2), 295-300.<br>5. Putkaradze, N., Litzenburger, M., Abdulmughni, A., Milhim, M., Brill, E., Hannemann, F., & Bernhardt, R. (2017). CYP109E1 is a novel versatile statin and terpene oxidase from Bacillus megaterium. Applied Microbiology and Biotechnology, 101, 8379-8393.
CYP109B1	4RM4	Girhard, M., Klaus, T., Khatri, Y., Bernhardt, R., & Urlacher, V. B. (2010). Characterization of the versatile monooxygenase CYP109B1 from Bacillus subtilis. Applied microbiology and biotechnology, 87, 595-607.
*Ba*CYP106A6	8HG9	Kim, K. H., Do, H., Lee, C. W., Subedi, P., Choi, M., Nam, Y., ... & Oh, T. J. (2022). Crystal structure and biochemical analysis of a cytochrome P450 steroid hydroxylase (BaCYP106A6) from Bacillus species. Journal of Microbiology and Biotechnology, 33(3), 387.

Supplementary Note S4. Biological experiment with uncurated bacterial enzymes.

Dr. Nelson named CYP154C9, CYP107G9, CYP1047A9, CYP106A4, and CYP106A5 through sequence blast (see Table S6). Each CYP was inserted in the pET28a vector and introduced into Escherichia coli C41(DE3). CYP was incubated in Luria–Bertani (LB) broth supplemented with antibiotics (100 μg/ml of ampicillin) at 37°C. A seed culture of CYP was grown in LB and maintained until an optical density at 600nm (OD600) of 0.6-0.8 was reached. 0.5 mM of FeCl3·6H2O and 1 mM of 5-aminolevulinic acid hydrochloride (5-ALA) were added to assist in heme formation. After cooling to 20°C, CYP was overexpressed by injecting 0.5 mM IPTG and incubated for 3 days. The harvested cell was suspended in a 50 mM potassium buffer (pH 7.4). The cell extract was sonicated to obtain the soluble protein solution. The protein solution was mixed with His-tag resin solution and eluted with 20-, 100-, and 250-mM imidazole potassium phosphate buffer. The eluted fraction containing the protein was concentrated using an Ultra centrifugal filter (Millipore, Ireland).

For the *in vitro* assay, steroid substrates (4-androstenedione, progesterone, and nandrolone) were used. A stock solution (100 mM) of the substrate was dissolved in DMSO and stored until use. The *in vitro* reaction was carried out in a total volume of 250 μl in a 50-mM potassium buffer (pH 7.4) consisting of 3 μM CYP, 2.5 mM iodo-benzene, and 200 μM steroid substrates. The CYP154C9, CYP107G9, and CYP1047A9 used iodo-benzene for the electron transfer system. The 10- μM CYP106A4 and A5 reacted with 100- μM FdX, 0.1U FdR, 200- μM steroid, 5-mM $MgCl_2$, 100- μg/ml catalase, and NADH generation system (1- mM NADH, 1- U glucose-6-phosphate dehydrogenase (G6P-DH), and 10- mM glucose-6-phosphate) in a 50- mM potassium buffer (pH 7.4). The reaction was initiated by adding 1 mM of NADH. Reaction mixtures were incubated at 30°C for 2 h, extracted twice with an equal volume of ethyl acetate, and dried completely. The dried samples were dissolved in methanol for HPLC analysis. HPLC analysis conditions were described previously [1].

Table S6. Strain of microorganisms and the CYP family for each strain of CYP used in this study

CYP Name	Species	Strain or isolated area
CYP154C9	Streptomyces alboniger	KCCM[a] 12598
CYP1047A9	Streptomyces sp.	KCCM 40463
CYP107G9	Actinomycete sp.	ATCC[b] 53650
CYP106A4	Paenibacillus sp.	Antarctica
CYP106A5	Paenibacillus sp.	Antarctica

[a]KCCM: Korean Culture Center of Microorganisms

[b]ATCC: American Type Microbiology

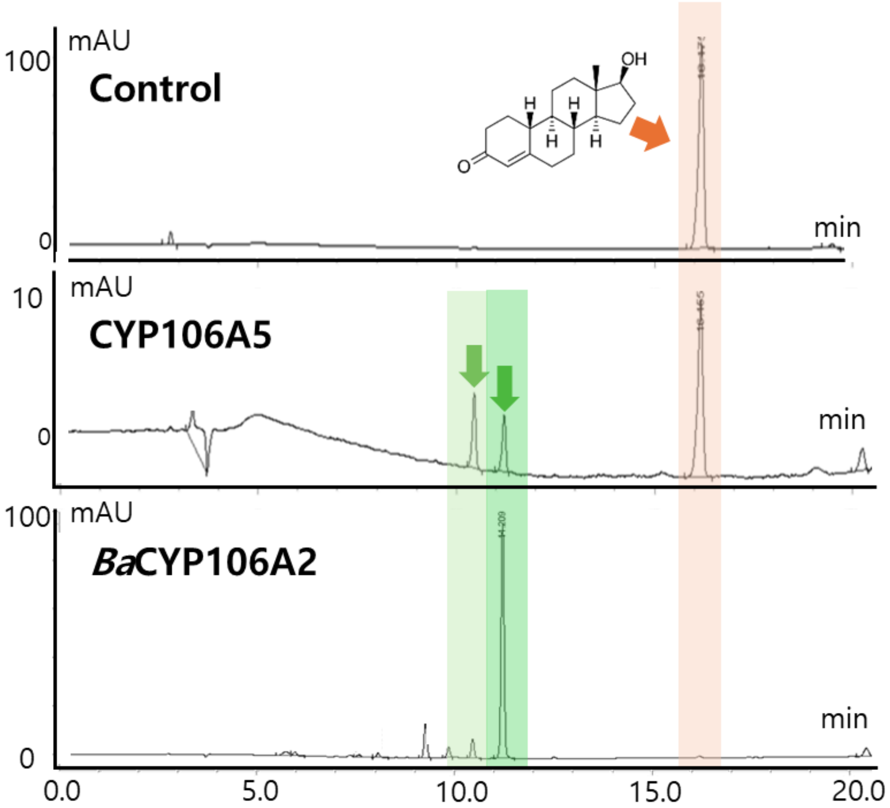

Figure S2. **HPLC chromatogram for comparison of *in vitro* assay results.** It used CYP106A5 and *Ba*CYP106A2 as target proteins and nandrolone as a ligand compound [1]. The orange arrows and boxes represent the nandrolone peaks, and the two green arrows and boxes represent the products, respectively. The main product of *Ba*CYP106A2 is 15β hydroxy compound.

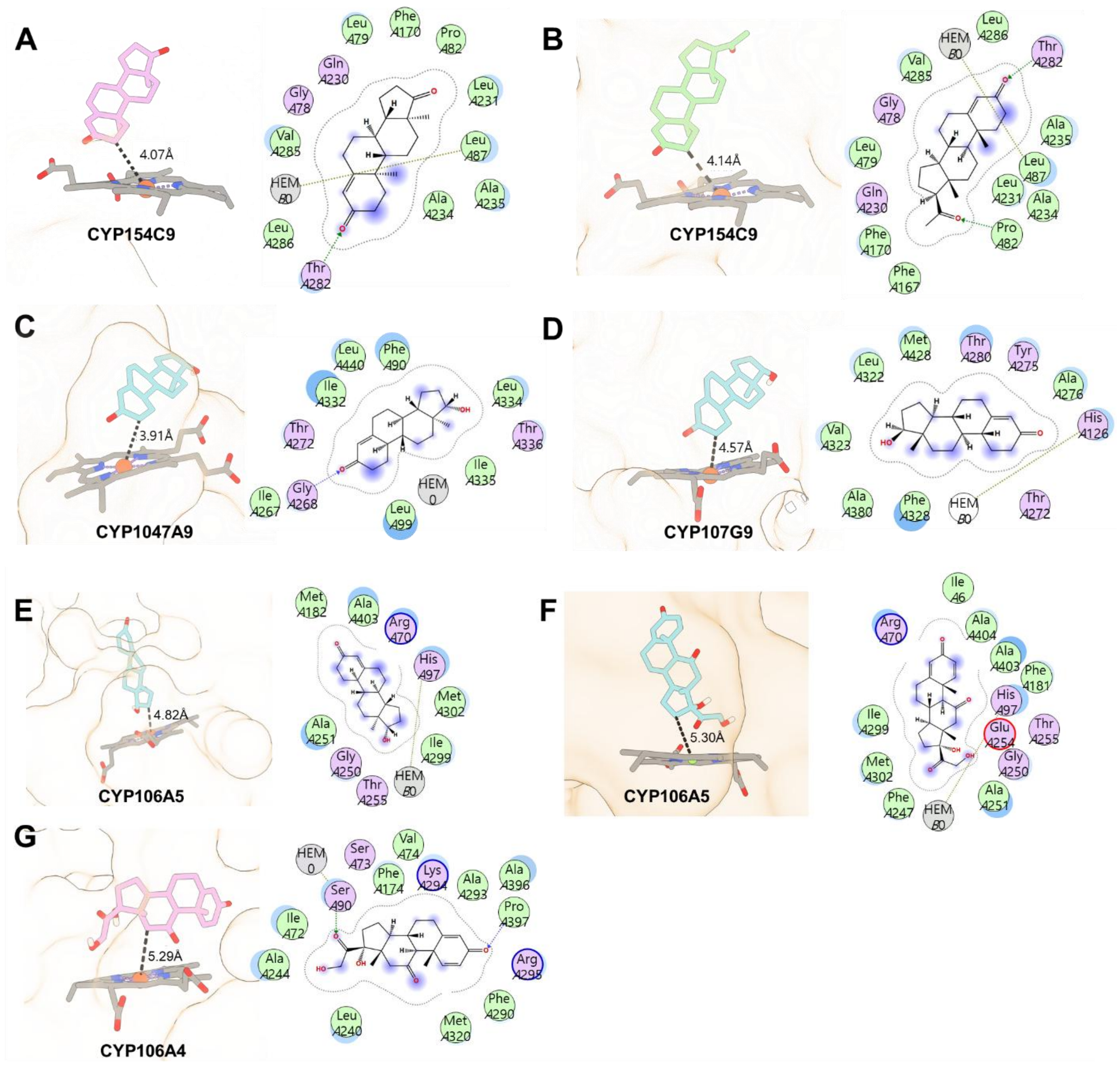

Figure. S3. Binding poses in target protein and 2D molecular docking diagrams showing the binding interactions of the best docked ligand compounds 4-androstenedione (A), progesterone (B), nandrolone (C, D and E), and prednisone (F and G).

Supplementary Note S5. Biophysical experiment with uncurated bacterial enzymes.

We also performed molecular docking to further assess the potential protein-ligand interactions. Since the three-dimensional structures of cytochrome P450 (CYP) protein strains are not available in the Protein Data Bank, the Chai Discovery web server [2] was used to generate homology models for all CYP isoforms. The pTM scores of all the homology models, along with the corresponding amino acid sequence lengths, are provided in Table S7. All ligand structures were downloaded from the PubChem database in SDF format and converted to PDBQT format by using Meeko v0.6.1 Python tool [https://github.com/forlilab/Meeko]. The grid box was centered on the heme iron of the CYP proteins, with a dimension of 18 × 18 × 18 Å. Molecular docking was performed using AutoDock Vina version 1.2.3 [3,4]. The docking scores and the distance between the heme iron and the nearest carbon atom are reported in Table 1. Snapshots of the protein-ligand docking were captured using ChimeraX [5], and ligand-protein interactions were analyzed by using Molecular Operating Environment (MOE) 2024.06 [6].

Table S7. Homology model (*pTM*) scores and amino acid lengths of the CYP Isoforms

CYP Isoforms	pTM score	Amino acid length
CYP154C9	0.9616	404
CYP1047A9	0.9417	455
CYP107G9	0.9163	439
CYP106A4	0.9592	410
CYP106A5	0.9333	418

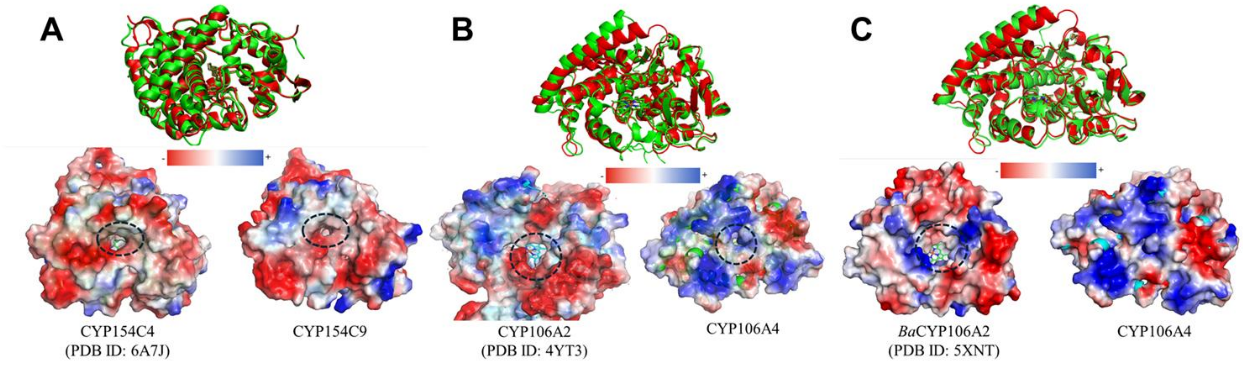

Supplementary Fig. S4. Superposition and electrostatic surface analysis of CYP154C9 and CYP106A4 with their isoform proteins. (A) CYP154C9, which were built by homology modeling, are shown in green, while CYP154C4, whose structures have been confirmed, are shown in red. In CYP154C4, a prominent tunnel-like cavity, highlighted in black, accommodates a small green substrate analog. (B) CYP106A4, constructed by homology modeling, is shown in green, and CYP106A2, whose structure has been determined, is shown in red. (C) Superposition results of CYP106A4 in green and BaCYP106A2 in red. Black lines indicate the entrances to the active sites.

Table S8. CPI scores predicted by PUCPI for five uncurated CYP proteins and three steroids. The reactions were validated through biological (HPLC) and biophysical (AutoDock) experiments.

Experiment No.	CYP	Substrate	Biological/biophysical assessment		PUCPI				
			HPLC	Biophysical assessment (Binding chance, Vina Score (kcal/mol), Nearest carbon (Dis. A)	TransformerCPI (θ=.46)	CNN-GNN (θ=.46)	SSNet (θ=.47)	MulinforCPI (θ=.47)	SVM (θ=.47)
Exp. 1	CYP154C9	4-Androstenedione	Pos	Pos (-6.35, C2 (4.07))	Pos (0.75)	Pos (0.67)	Pos (0.61)	Pos (0.795)	Neg (0.293)
Exp. 2	CYP154C9	Progesterone	Pos	Pos (-7.19, C2 (4.14))	Pos (0.64)	Neg (0.27)	Neg (0.26)	Neg (0.228)	Neg (0.428)
Exp. 3	CYP154C9	Nandrolone	Pos	Pos (-8.27, C2 (4.49))	Pos (0.51)	Neg (0.04)	Pos (0.48)	Pos (0.524)	Neg (0.415)
Exp. 4	CYP1047A9	Nandrolone	Pos	Pos (-8.24, C2 (3.91))	Pos (0.48)	Pos (0.61)	Neg (0.47)	Neg (0.427)	Neg (0.294)
Exp. 5	CYP107G9	Nandrolone	Pos	Pos (-7.66, C2 (4.57))	Pos (0.72)	Pos (0.71)	Neg (0.42)	Neg (0.214)	Pos (0.986)
Exp. 6	CYP106A4	Nandrolone	Pos	Pos (-8.48, C15 (4.69))	Pos (0.47)	Neg (0.23)	Neg (0.11)	Pos (0.716)	Pos (0.866)
Exp. 7	CYP106A5	Nandrolone	Pos	Neg (-6.61, C16 (4.82))	Pos (0.70)	Neg (0.21)	Pos (0.93)	Pos (0.972)	Pos (0.993)
Exp. 8	CYP106A4	Prednisone	Neg	Neg (-6.31, C12 (5.29))	Neg (0.256)	Neg (0.133)	Pos (0.824)	Pos (0.491)	Neg (0.170)

Exp. 9	CYP106A5	Prednisone	Neg	Neg (-6.15, C16 (5.29))	Neg (0.417)	Pos (0.617)	Pos (0.604)	Neg (0.236)	Neg (0.196)

Table S9. CPI scores predicted by PU-Contrastive for five uncurated CYP proteins and three steroids. The reactions were validated through biological (HPLC) and biophysical (AutoDock) experiments.

Experiment No.	CYP	Substrate	Biological/biophysical assessment		PU-Contrastive			
			HPLC	Biophysical assessment (Binding chance, Vina Score (kcal/mol), Nearest carbon (Dis. A)	TransformerCPI (θ=.47)	CNN-GNN (θ=.47)	SSNet (θ=.47)	MulinforCPI (θ=.48)
Exp. 1	CYP154C9	4-Androstenedione	Pos	Pos (-6.35, C2 (4.07))	Pos (0.847)	Pos (0.651)	Pos (0.72)	Pos (0.547)
Exp. 2	CYP154C9	Progesterone	Pos	Pos (-7.19, C2 (4.14))	Pos (0.87)	Pos (0.52)	Neg (0.31)	Pos (0.514)
Exp. 3	CYP154C9	Nandrolone	Pos	Pos (-8.27, C2 (4.49))	Pos (0.62)	Neg (0.11)	Pos (0.74)	Pos (0.612)
Exp. 4	CYP1047A9	Nandrolone	Pos	Pos (-8.24, C2 (3.91))	Pos (0.48)	Neg (0.42)	Neg (0.34)	Neg (0.428)
Exp. 5	CYP107G9	Nandrolone	Pos	Pos (-7.66, C2 (4.57))	Pos (0.89)	Pos (0.81)	Neg (0.35)	Pos (0.518)
Exp. 6	CYP106A4	Nandrolone	Pos	Pos (-8.48, C15 (4.69))	Pos (0.78)	Neg (0.25)	Neg (0.21)	Pos (0.654)
Exp. 7	CYP106A5	Nandrolone	Pos	Neg (-6.61, C16 (4.82))	Pos (0.71)	Neg (0.19)	Pos (0.92)	Pos (0.982)

Exp. 8	CYP106A 4	Prednisone	Neg	Neg (-6.31, C12 (5.29))	Neg (0.26)	Neg (0.231)	Pos (0.822)	Neg (0.401)
Exp. 9	CYP106A 5	Prednisone	Neg	Neg (-6.15, C16 (5.29))	Neg (0.355)	Pos (0.728)	Pos (0.677)	Neg (0.233)

References

1. Kim KH, Lee CW, Dangi B, Park SH, Park H, Oh TJ, Lee JH. Crystal Structure and Functional Characterization of a Cytochrome P450 (BaCYP106A2) from Bacillus sp. PAMC 23377. J Microbiol Biotechnol. 2017 Aug 28;27(8):1472-1482. doi: 10.4014/jmb.1706.06013. PMID: 28633515.
2. C. Discovery, J. Boitreaud, J. Dent, M. McPartlon, J. Meier, V. Reis, A. Rogozhnikov, K. Wu, Chai-1: decoding the molecular interactions of life, bioRxiv (2024), https://doi.org/10.1101/2024.10.10.615955 (2024.10.10.615955).
3. Trott, O. and Olson, A.J., 2010. AutoDock Vina: improving the speed and accuracy of docking with a new scoring function, efficient optimization, and multithreading. *Journal of computational chemistry*, *31*(2), pp.455-461.
4. Eberhardt, J., Santos-Martins, D., Tillack, A.F. and Forli, S., 2021. AutoDock Vina 1.2. 0: New docking methods, expanded force field, and python bindings. *Journal of chemical information and modeling*, *61*(8), pp.3891-3898.
5. Meng, E.C., Goddard, T.D., Pettersen, E.F., Couch, G.S., Pearson, Z.J., Morris, J.H. and Ferrin, T.E., 2023. UCSF ChimeraX: Tools for structure building and analysis. *Protein Science*, 32(11), p.e4792.
6. Molecular Operating Environment (MOE), 2024.0601 Chemical Computing Group ULC, 910-1010 Sherbrooke St. W., Montreal, QC H3A 2R7, 2025.
